# Integrative phytochemical profiling and *in silico* nutrigenomic predictions of Chinese tea–Saudi *Mentha longifolia* blend formulations

**DOI:** 10.3389/fnut.2026.1753616

**Published:** 2026-03-27

**Authors:** Thorya A. Fallatah, Hala M. Abdelmigid, Amal A. Alyamani, Maissa M. Morsi, Mohammed Ali, Jingmei Lu, Jian Zhao, Yasmin M. Heikal

**Affiliations:** 1 Department of Biological Sciences, University of Jeddah, Jeddah, Saudi Arabia; 2 Department of Biotechnology, College of Science, Taif University, Taif, Saudi Arabia; 3 Biology Department, College of Science, Taif University, Taif, Saudi Arabia; 4 Maryout Research Station, Genetic Resources Department, Desert Research Center, Cairo, Egypt; 5 College of Plant Science, Jilin University, Changchun, China; 6 National Research Center of Engineering and Technology for Utilization of Botanical Functional Ingredients, Hunan Agricultural University, Changsha, Hunan, China; 7 State Key Lab of Tea Plant Biology and Utilization, College of Tea and Food Science & Technology, Anhui Agricultural University, Anhui, China; 8 Botany Department, Faculty of Science, Mansoura University, Mansoura, Egypt

**Keywords:** Chinese green tea, special flavor herb tea, nutrigenomics, functional genomics, eucalyptol, sesquiterpenes, phenolic compounds, herbal blends

## Abstract

Medicinal plants represent valuable sources of bioactive compounds with therapeutic and economic potential. In Saudi Arabia, *Mentha longifolia* L. has long been used in traditional medicine, while China is renowned for its diverse teas derived from *Camellia sinensis* L. However, the tissue-specific genomic impact of their metabolites remains poorly understood. We tested three replicates of each blending ratio: 1:1, 1:2, and 2:1 (Tea:*Mentha*). Moreover, we investigated the putative expression profiles of 273 genes across human tissues using transcriptomic databases to explore the nutrigenomic effects of tea–*Mentha* blends. 1:2 (Tea:*Mentha*) Replicate 2 was dominated with 50.77% bioactive compounds, making it the strongest candidate overall. The highest *Mentha* aroma and bioactive compounds included eucalyptol (~17.3%), (+)-2-bornanone (~12.2%), n-hexadecanoic acid (~16.6%), and phytol (~3.2%). Lipid-derived molecules, including phytol, oleamide, and linolenic acids, showed the strongest transcriptional activation, particularly in endocrine and reproductive tissues, whereas alkaloids such as caffeine exhibited moderate effects. The preliminary integrative analysis combining experimental phytochemical profiling with computational nutrigenomic predictions is designed to generate testable hypotheses for future functional assays. Such blends may contribute to product diversification, standardization, and quality enhancement in the herbal tea industry.

## 1 Introduction

Medicinal plants (MPs) have long been used to treat ailments in both humans and animals in Saudi Arabia and across the Arabian Peninsula. The flora in this region is a rich source of MPs, with some species being endemic (1–3). A study of 612 participants revealed that 48.9, 33.7, and 25.3% of individuals used herbal medicine during pregnancy, labor, and postpartum, respectively, for purposes such as cleansing the womb, facilitating labor, and enhancing overall health (4).

Green tea (*Camellia sinensis*) is among the most widely consumed herbal beverages worldwide and is celebrated for its antioxidant and health-promoting properties (5–7). Its bioactive components include phenols, alkaloids, flavonoids, tannins, steroids, and minerals, with epigallocatechin gallate (EGCG) being the most potent compound (8–10). Tea catechins exhibit a broad spectrum of pharmacological effects, including anti-inflammatory (11), anti-arthritic (12), anticarcinogenic (13), anticancer (14), antibacterial (15), antiviral (16), antifungal (17), anticoccidial (18), antiprotozoal (19), antiparasitic (20), anti-infective (21), hypocholesterolemic (22), vasoprotective (23), and hypolipidemic activities (24). Caffeine, another key component, serves as a central nervous system stimulant that boosts alertness and reduces fatigue (25). Furthermore, clinical studies have shown that regular green tea consumption reduces oxidative DNA damage and lipid peroxidation (26, 27).

Although green tea is renowned for its health benefits, it frequently lacks sensory appeal, prompting the practice of blending it with herbs to enhance its flavor and functionality. These blends can improve nutrient distribution and augment anti-inflammatory and antioxidant properties while mitigating cytotoxicity (25). Among the promising candidates for blending is *Mentha longifolia* (L.) Hudson, commonly referred to as wild mint, which is extensively cultivated in the Madinah region (28, 29). Traditionally consumed as a refreshing tea or food additive (30), *Mentha* species are rich in essential oils, polyphenols, flavonoids, and coumarins, and have demonstrated antioxidant, antibacterial, antifungal, antiviral, and anticancer properties (31–36). Tea consumption in Saudi Arabia is remarkably widespread, with annual imports exceeding 31,000 tonnes and exports valued at USD 10.9 million in 2022. The market is projected to grow by 2.1% in volume by 2025 (37–39). Sensory quality, including flavor, aroma, texture, and appearance, plays a vital role in determining consumer acceptance (40, 41). Research suggests that blending herbal extracts in different ratios can improve sensory properties, with taste and flavor being the primary factors influencing consumer preferences (42–44).

Plant metabolites, such as flavonoids, phenols, glucosinolates, and organic acids, play a crucial role in plant defense mechanisms and human health, offering properties such as antioxidant, anticancer, antihypertensive, antibacterial, and immune modulation (45). Tea contains over 4,000 bioactive compounds, including catechins, theanine, caffeine, and terpenes, which together enhance its flavor and health benefits (46–49). Recent advancements in metabolomics and transcriptomics have deepened our understanding of the biosynthesis and regulation of these secondary metabolites (50). Genome assembly of *C. sinensis* has revealed high expression levels of genes involved in flavonoid and caffeine biosynthesis, with catechin accumulation linked to the phenylpropanoid pathway (51–54). Current breeding efforts focus on developing germplasm with increased catechin content and reduced caffeine levels to meet consumer preferences (55).

*M. longifolia* is rich in bioactive compounds, including menthol and terpenes, which hold considerable industrial and pharmacological significance (56, 57). Advanced transcriptomic and metabolomic studies have elucidated the regulatory mechanisms governing anthocyanin and flavonoid biosynthesis (58). Despite extensive research on the pharmacological properties of tea and *Mentha*, the tissue-specific transcriptional effects of their phytochemicals in humans remain largely unexplored.

Several classes of nutrients and phytochemicals commonly present in tea and aromatic herbs—including catechins, polyphenols, terpenoids, and fatty acids—are known to influence gene networks involved in antioxidant defense, xenobiotic detoxification, lipid metabolism, and cellular stress responses. Previous nutrigenomic studies have shown that compounds such as epigallocatechin gallate, quercetin, and dietary fatty acids can modulate the expression of genes associated with the Nrf2 antioxidant pathway, phase II detoxification enzymes, inflammatory mediators, and metabolic regulators (e.g., *CAT*, *HMOX1*, *GPX1*, *NQO1*, and *PPARG*) (22). These interactions are typically mediated through signaling cascades or transcription factor activation, providing a conceptual basis for exploring how phytochemicals may be associated with tissue-specific gene expression patterns. In the present study, such transcriptomic analyses are used purely in a predictive and hypothesis-generating manner to contextualize the potential molecular relevance of the GC-MS-identified volatile phytochemicals.

The present study aimed to characterize the volatile phytochemical profiles of Chinese green tea (*Camellia sinensis*) blended with *Mentha longifolia* using GC-MS, and to integrate these experimental findings with predictive, database-derived *in silico* nutrigenomic analyses. By mapping GC-MS-detected phytochemicals to curated compound–gene associations and baseline tissue expression datasets, we explored the putative molecular pathways and gene networks potentially connected to these compounds. This integrative approach is intended to generate hypotheses regarding the phytochemical interactions of tea–*Mentha* blends and to support the future development of novel formulations with distinctive chemical characteristics and potential functional relevance.

## 2 Materials and methods

### 2.1 Plant material and a new tea herbal product

Dry leaves from two medicinal plant species were collected for the preparation of herbal tea blends. The first species, green tea (*Camellia sinensis*), was obtained from Huazhong Agricultural University, Wuhan, Hubei Province, China, in March 2025. The second species, *Mentha longifolia* (wild mint), was collected from Taif city, Makkah region of Saudi Arabia, in April 2025. All plant materials were processed, dried, and stored for no longer than 6 months prior to analysis, ensuring that the green tea remained well within its acceptable shelf-life. Both materials were stored in airtight, light-protected containers at 4 °C to prevent degradation of volatile constituents. Only samples confirmed to be within this controlled storage period were used in GC-MS profiling.

In total, five tea formulations were prepared: two single-herb teas (*C. sinensis* and *M. longifolia*) and three blends with different mixing ratios: (1:1), (1:2), and (2:1) of *C. sinensis* to *M. longifolia*. Mixing ratios were expressed as volume per gram (V/g:V/g). Each formulation, whether single-herb or blended, was packed into biodegradable non-woven polylactic acid (PLA) tea bags. Each tea bag contained approximately 1.5 g of dried plant material.

### 2.2 Extraction and analysis of phytochemicals and metabolites

The extraction and analysis of phytochemical and metabolite compounds from the original and blended herbal tea samples were performed following the procedures described by Ali et al. (59–64). Briefly, dried samples were soaked in n-hexane in 60 mL bottles and incubated with shaking at 29 °C and 175 rpm for 70 h. The extracts were then centrifuged at 4,500 rpm for 11 min at 4 °C to remove plant debris. The clarified solvent was concentrated and transferred into 1.5 mL amber glass crimp vials with screw tops.

GC-MS analysis was performed using a Shimadzu GCMS-QP2010 Ultra system equipped with an RTX-5MS capillary column (30 m × 0.25 mm i.d., film thickness 0.25 μm). Helium was used as the carrier gas at a constant flow rate of 1.0 mL/min. The injector temperature was set to 250 °C, with a split ratio of 1:10. The oven temperature program was: initial 60 °C (held 2 min), ramped at 3 °C/min to 180 °C, then 10 °C/min to 280 °C, held for 10 min. The interface temperature was 280 °C, and the ion source temperature was 230 °C. Mass spectra were acquired in EI mode at 70 eV over a scan range of *m*/*z* 40–500. Compound identification was achieved by matching mass spectra with the NIST (2014), Wiley (10th edition), and Volatile Organic Compounds (VOC) libraries, using a similarity threshold of ≥85%. Retention indices and literature comparisons were used to further validate compound identity, as described previously (59–64).

GC-MS quality control procedures included the use of solvent blanks, instrument blanks, and non-loaded PLA tea-bag blanks to detect background artefacts, column bleed, or environmental contaminants. Only peaks consistently present in sample replicates and absent in all control blanks were considered authentic plant-derived compounds. Phthalates, siloxanes, xylenes, and chlorinated hydrocarbons were identified as laboratory or analytical contaminants and therefore removed from all biological interpretation and downstream analyses.

### 2.3 Identification and assignment of genes associated with phytochemical compounds

The GC-MS analysis represents the only experimental and empirically validated component, providing direct chemical characterization of the tea-*Mentha* blends through established analytical procedures. In contrast, the transcriptomic mapping relies exclusively on non-experimental, database-derived predictive analyses, using curated chemical–gene associations and publicly available baseline tissue expression datasets that do not reflect compound exposure, bioavailability, or physiological responses. The baseline tissue expression profiles from public transcriptomic was occurred through the Comparative Toxicogenomics Database (CTD).^1^ Using the CTD chemical–gene interaction tools, the top 10 interacting genes were retrieved for each phytochemical compound. No compound exposure or dose–response analysis was performed. The GC-MS results reflect chemical composition only, not physiological activity. No *in vivo*, *in vitro*, or clinical validation was performed. Therefore, the integration of these two layers of information is intended solely as hypothesis-generation rather than biological inference, serving to identify potential molecular targets and guiding future functional validation rather than suggesting any confirmed nutrigenomic or systemic effects of the blends.

#### 2.3.1 Putative expression patterns of target genes in human circulatory and respiratory tissues

Putative tissue-specific expression profiles of 273 human genes were extracted from transcript expression databases covering 36 tissues of the circulatory and respiratory systems. The analyzed tissues included: CD33^+^ myeloid cells [1], fetal lung [1], heart [1], blood [1], skin [1], bronchial epithelial cells [1], CD71^+^ early erythroid cells [1], kidney [1], lung [1], trachea [1], placenta [1], ventricle [5], saphenous vein [5], urethra [5], bronchus [5], kidney medulla [5], atrium [5], coronary artery [5], lung [5], kidney cortex [5], trachea [5], heart [4], kidney [4], skin [4], fetal lung [4], bladder [4], lung [4], trachea [4], placenta [4], pulmonary trunk [7], pulmonary vessels [7], pleura [2], aorta [8], vena cava [8], diaphragm [3], and bronchioles [6]. Expression visualization was performed using the Human Electronic Fluorescent Pictograph (eFP) browser (https://bar.utoronto.ca/efp_human/cgi-bin/efpWeb.cgi, accessed on 25 October 2025). This platform enabled comparative mapping of gene activity across tissues, providing insights into the potential tissue-specific transcriptional roles of phytochemical-predictive associated genes (65–72).

#### 2.3.2 Putative tissue expression patterns of target genes based on the Illumina Body Map 2—FPKM

Putative tissue-specific expression profiles of 273 human genes were extracted from the Illumina Body Map 2 transcript expression database, quantified as fragments per kilobase of exon model per million mapped reads (FPKM). Data were obtained across 16 male and female tissues, including: adrenal gland (male), liver (male), white blood cells (male), skeletal muscle (male), lung (male), heart (male), prostate (male), testes (male), thyroid (female), breast (female), ovary (female), brain (female), lymph (female), kidney (female), colon (female), and adipose tissue (female). Expression profiles were visualized using the Human Electronic Fluorescent Pictograph (eFP) browser (https://bar.utoronto.ca/efp_human/cgi-bin/efpWeb.cgi?dataSource=Illumina_Body_Map_2_-_FPKM&mode=Absolute&primaryGene=CCR5&secondaryGene=PAX6&useThreshold=&thres old = 334.81& grey_low = None &grey_stddev = None), accessed on 20 October 2025. This approach enabled the comparative mapping of gene activity across male and female tissues, providing insights into the potential tissue-specific transcriptional roles of phytochemical-associated genes (65–72).

#### 2.3.3 Putative expression patterns of target genes in human nervous system tissues

Putative tissue-specific expression profiles of 273 human genes were extracted from transcript expression databases covering 57 tissues of the nervous system. The analyzed regions included: whole brain [3], corpus callosum [3], amygdala [3], fetal brain [3], thalamus [3], caudate nucleus [3], hippocampus [3], cerebellum [3], spinal cord [3], ciliary ganglion [1], amygdala [1], subthalamic nucleus [1], fetal brain [1], thalamus [1], trigeminal ganglion [1], whole brain [1], olfactory bulb [1], caudate nucleus [1], cingulate cortex [1], globus pallidus [1], hypothalamus [1], prefrontal cortex [1], parietal lobe [1], occipital lobe [1], temporal lobe [1], cerebellum [1], cardiac myocyte [1], atrioventricular node [1], dorsal root ganglion [1], cerebellar peduncle [1], pons [1], medulla oblongata [1], spinal cord [1], SCG [1], corpus callosum [2], amygdala [2], subthalamic nucleus [2], hippocampus [2], thalamus [2], nucleus accumbens [2], trigeminal ganglion [2], hypothalamus [2], putamen [2], substantia nigra [2], cerebral cortex [2], frontal lobe [2], parietal lobe [2], occipital lobe [2], temporal lobe [2], cerebellum [2], dorsal root ganglion [2], midbrain [2], VTA [2], SVN [2], medulla oblongata [2], spinal cord [2], and nodose nucleus [2]. Expression visualization was performed using the Human Electronic Fluorescent Pictograph (eFP) browser (https://bar.utoronto.ca/efp_human/cgi-bin/efpWeb.cgi?dataSource=Nervous, accessed on 5 October 2025). This platform enabled comparative mapping of gene activity across central and peripheral nervous system tissues, providing insights into the potential neuro-specific transcriptional roles of phytochemical-predictive-associated genes (65–72).

#### 2.3.4 Putative expression patterns of target genes in human skeletal, immune, and digestive tissues

Putative tissue-specific expression profiles of 273 human genes were extracted from the skeletal, immune, and digestive transcript expression database, covering 58 tissues. The analyzed tissues included: adipocyte [1], smooth muscle [1], psoas muscle [1], salivary gland [1], CD56^+^ NK cells [1], CD19^+^ B cells [1], CD4^+^ T cells [1], BDCA4^+^ dendritic cells [1], CD14^+^ monocytes [1], CD105^+^ endothelial cells [1], appendix [1], bone marrow [1], CD8^+^ T cells [1], 721 B lymphocytes [1], pancreas [1], lymph node [1], thymus [1], CD34^+^ cells [1], tonsil [1], tongue [1], islet cells [1], liver [1], fetal liver [1], adipose tissue omental [3], adipose tissue subcutaneous [3], adipose tissue [3], skeletal muscle [3], bone marrow [3], salivary gland [3], esophagus [3], cardiac stomach [3], liver [3], spleen [3], pharyngeal mucosa [3], fundus [3], pylorus [3], lymph node [3], cecum [3], tonsil [3], oral mucosa [3], tongue [3], superior tongue [3], skeletal muscle [4], bone marrow [4], liver [4], fetal liver [4], salivary gland [4], stomach [4], colon [4], small intestine [4], thymus [4], pancreas [4], spleen [4], pelvis [7], vertebral column [7], large intestine [2], lymph vessels [6], and pancreatic duct [5]. Expression profiles were visualized using the Human Electronic Fluorescent Pictograph (eFP) browser (https://bar.utoronto.ca/efp_human/cgi-bin/efpWeb.cgi?dataSource=Skeletal_Immune_Digestive, accessed on 5 October 2025). This analysis enabled comparative mapping of gene activity across skeletal, immune, and digestive tissues, providing insights into the potential roles of phytochemical-predictive-associated genes (65–72).

#### 2.3.5 Putative expression patterns of target genes in human reproductive system tissues

Putative tissue-specific expression profiles of 273 human genes were extracted from the reproductive system transcript expression database, covering 38 tissues. The analyzed tissues included: pituitary [2], vulva [2], vagina [2], prostate [2], nipple [2], testes [2], mammary gland [2], cervix [2], hypothalamus [2], ovary [2], thyroid [2], adrenal cortex [2], myometrium [2], endometrium [2], pituitary [1], testes [1], adrenal gland [1], prostate [1], Leydig cells [1], pancreas [1], hypothalamus [1], thyroid [1], adrenal cortex [1], fetal thyroid [1], germ cells [1], seminiferous tubule [1], uterus corpus [1], uterus [1], ovary [1], prostate [3], uterus [3], bladder [3], testes [3], pancreas [3], breast [3], thyroid [3], ovary [3], and pituitary [3]. Expression profiles were visualized using the Human Electronic Fluorescent Pictograph (eFP) browser (https://bar.utoronto.ca/efp_human/cgi-bin/efpWeb.cgi?dataSource=Reproductive, accessed on 1 October 2025). This analysis enabled comparative mapping of gene activity across male and female reproductive tissues, providing insights into the potential roles of phytochemical-predictive-associated genes in reproductive physiology and regulation (65–72).

### 2.4 Statistical analysis

For GC/MS analysis, three biological replicates were used to ensure reproducibility. Phytochemical profiles were visualized through multiple approaches, including Venn diagrams, bar charts, heatmaps, pie charts, and scatter plots, all generated using Plotly, a Python-based graphing library designed for interactive and publication-quality visualizations (Plotly.js, Version 3.1.0) (73). For tissue-specific gene expression analysis, all data processing and visualizations were conducted in Jupyter Notebook using Python 3.12. Several Python libraries were employed: Plotly Express^2^ for interactive scatter, bar, and Sankey plots; Matplotlib^3^ for constructing base heatmaps and customizing color scales; Seaborn^4^ for high-level heatmap plotting with built-in palettes and annotations; Pandas^5^ for handling gene expression data in tabular form; and NumPy^6^ for numerical operations and matrix handling. This integrated workflow ensured robust statistical treatment of GC/MS data and comprehensive visualization of gene expression patterns across tissues.

## 3 Results

### 3.1 GC/MS of Chinese tea and *Mentha longifolia*

#### 3.1.1 Formulation of blending: replicates, selection, and ranking

The GC-MS analysis of Chinese tea and *Mentha longifolia* blends revealed substantial variability in compound diversity, category distribution, and cumulative peak area across different blending ratios (1:1, 1:2, and 2:1). Ranking of replicates based on the combined percentage peak area of key compounds highlighted clear differences among formulations (Supplementary Figure S1A). The tea-dominant blend 2 Tea + 1 *Mentha* 1 achieved the highest cumulative peak area (57.64%) but exhibited low diversity (22 compounds), indicating enrichment of a few dominant metabolites. In contrast, the *Mentha*-rich blend 1 Tea + 2 *Mentha* 1 recorded the highest diversity (116 compounds) with a peak area of 50.24%, making it the most phytochemically rich formulation. Balanced blends such as 1 Tea + 1 *Mentha* 3 showed moderate diversity (65 compounds) and peak area (~95%), suggesting suitability for formulations targeting both functional and sensory attributes (Supplementary Figure S1B).

Scatter plot analysis confirmed these trends, showing that increasing *Mentha* proportion enhanced compound diversity, whereas tea-rich blends clustered toward higher peak area but lower diversity. Diversity scores ranged from 22 to 116, with 1 Tea + 2 *Mentha* 1 emerging as the most promising replicate due to its broad phytochemical spectrum and balanced distribution of functional categories. Monoterpenes dominated most blends, particularly in 1 Tea + 2 *Mentha* 1 (49.02%) and 2 Tea + 1 *Mentha* 1 (59.59%), reflecting *Mentha*’s contribution of volatile terpenoids. Sesquiterpenes were abundant in 2 Tea + 1 *Mentha* 3 (37.05%), suggesting enhanced aromatic complexity in tea-rich blends, while fatty acids peaked in 1 Tea + 2 *Mentha* 2 (43.95%), potentially contributing to antioxidant and emulsifying properties. Alcohols and siloxanes were present in smaller proportions, with alcohols reaching 29.43% in 1 Tea + 2 *Mentha* 3, indicating possible antimicrobial and preservative effects (Supplementary Figure S1C).

Collectively, 1 Tea + 2 *Mentha* 1 was identified as the most promising blend, combining the highest diversity (116 compounds) with a balanced phytochemical profile dominated by monoterpenes (49.02%), alcohols (13.69%), and fatty acids (7.43%). For the 1:1 ratio, 1 Tea + 1 *Mentha* 3 ranked best, with a diversity score of 65 and notable contributions from alcohols (22.05%) and sesquiterpenes (7.14%), suggesting synergistic antioxidant and antimicrobial potential. Tea-dominant blends (2:1) favored 2 Tea + 1 *Mentha* 1, which, despite low diversity, exhibited the highest monoterpene proportion (59.59%) and significant siloxane content (15.04%), potentially enhancing fragrance and oxidative stability.

**Figure 1:**
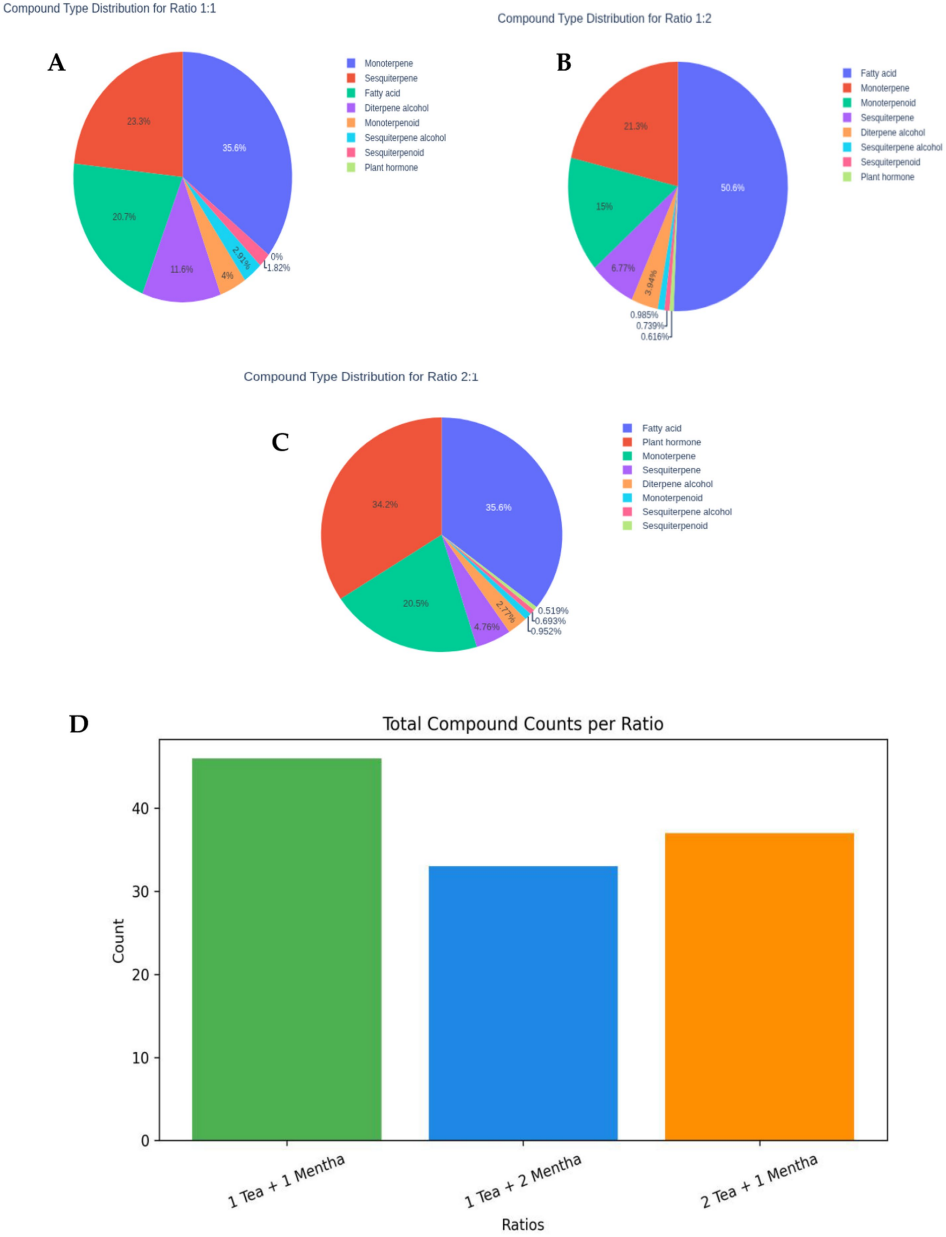
Comparative distribution of compound categories and total compound counts across three tea–*Mentha* ratios. The pie charts represent the percentage distribution of compound categories: **(A)** 1 Tea + 1 *Mentha* blend, **(B)** 1 Tea + 2 *Mentha* blend, **(C)** 2 Tea + 1 *Mentha* blend. Different colors represent compound types. **(D)** Bar chart compares the total number of identified compounds per ratio.

Compound type distribution further highlighted compositional shifts across blend ratios (Figures 1A–D). In 1 Tea + 1 *Mentha*, monoterpenes (35.6%) were predominant, followed by sesquiterpenes (23.3%) and fatty acids (20.7%), with diterpene alcohols (11.6%) and minor classes (<5%) contributing to the profile. In 1 Tea + 2 *Mentha*, fatty acids increased markedly (50.6%), while monoterpenes decreased to 21.3% and sesquiterpenes to 6.77%, reflecting a lipid-rich *Mentha* contribution. Conversely, 2 Tea + 1 *Mentha* showed a more balanced distribution, with fatty acids (35.6%) and sesquiterpenes (34.2%) dominating, and monoterpenes contributing 20.5%. Compound type distribution further highlighted compositional shifts across blend ratios (Figures 1A–D). In 1 Tea + 1 *Mentha*, monoterpenes (35.6%) were predominant, followed by sesquiterpenes (23.3%) and fatty acids (20.7%), with diterpene alcohols (11.6%) and minor classes (<5%) contributing to the profile. In 1 Tea + 2 *Mentha*, fatty acids increased markedly (50.6%), while monoterpenes decreased to 21.3% and sesquiterpenes to 6.77%, reflecting a lipid-rich *Mentha* contribution. Conversely, 2 Tea + 1 *Mentha* showed a more balanced distribution, with fatty acids (35.6%) and sesquiterpenes (34.2%) dominating, and monoterpenes contributing 20.5%.

#### 3.1.2 Top 10 compounds ranking in each blending ratio

The heatmap analysis demonstrated clear differences in the relative abundance of key bioactive compounds across the three tea–*Mentha* blending ratios (1:1, 1:2, and 2:1) (Supplementary Figure S2A). Eucalyptol increased steadily from 9.8% at the 1:1 ratio to 23.7% at 2:1, highlighting its dominance in tea-rich blends. Linoleic acid was the most consistent fatty acid, maintaining high levels of 24.5% in both 1:2 and 2:1 ratios, while palmitic acid remained stable at 16.6% in these blends. Camphor peaked at 12.2% in the 1:2 ratio but declined sharply in 2:1. Notably, methyl jasmonate was absent in 1:1, present only at trace levels in 1:2 (0.5%), and rose dramatically to 39.5% in 2:1, making it the most abundant compound in tea-dominant blends and a clear chemical marker distinguishing this ratio. Other constituents, such as caryophyllene, humulene, and phytol, remained relatively low and stable across all ratios, suggesting minor contributions to the whole profile.

Bar charts of compound contributions by retention time and scatter plots of retention time versus peak area (Supplementary Figures S2B–S4C) further emphasized the dominance of specific constituents. Among the top contributors, 9,12-octadecadienoic acid (Z, Z-) exhibited the highest peak area (24.56%) at a retention time of ~49.48 min in the 1 Tea + 2 *Mentha* blend, followed by eucalyptol (23.76%, R.T. 13.88) in 2 Tea + 1 *Mentha* and α-pinene (17.5%, R.T. 7.20) in 1 Tea + 2 *Mentha*. In addition, heneicosane (16.6%, R.T. 54.35) and α-bisabolol (16.12%, R.T. 48.43) were prominent in 1 Tea + 1 *Mentha*, reflecting substantial contributions from oxygenated compounds and terpenes (Supplementary Figure S3). Mostly, these results indicate a strong correlation between retention time and compound class distribution. Later-eluting compounds such as fatty acids and hydrocarbons dominated *Mentha*-rich blends, while early-eluting monoterpenes contributed significantly to aroma and antimicrobial properties. The observed variability underscores how blending ratios shape chemical complexity and functional potential, with each formulation offering a distinct balance of bioactive constituents (Table 1).

**Table 1:** Top 10 Compounds with common name, type, retention time (RT), %peak area, and blending ratio of Chinese tea and *Mentha* blends.

Ratio	Common name	Compound type	R.T.	% peak area
1 Tea + 1 *Mentha*	Heneicosane	Alkane (hydrocarbon)	54.35	16.6
	Alpha-bisabolol	Sesquiterpene alcohol	48.436	16.12
	Pentacosane	Alkane (hydrocarbon)	63.24	16.01
	Eugenol	Phenylpropanoid	74.673	14.45
	Longifolene	Sesquiterpene hydrocarbon	27.985	4.7
	Nerolidol	Sesquiterpene alcohol	39.959	3.85
	Phenylethyl alcohol	Aromatic alcohol	49.032	3.13
	Linoleic acid	Polyunsaturated fatty acid	47.9	3.09
	Alpha-pinene	Monoterpene	48.011	2.89
	Palmitic acid	Saturated fatty acid	18.149	2.71
1 Tea + 2 *Mentha*	p-Xylene	Aromatic hydrocarbon	49.483	24.56
	Beta-caryophyllene	Sesquiterpene	7.204	17.5
	Tetrachloroethane	Chlorinated hydrocarbon	45.333	16.56
	Phytol	Diterpene alcohol	5.575	9.46
	Beta-pinene	Monoterpene	29.823	3.8
	Gamma-muurolene	Sesquiterpene hydrocarbon	6.706	3.45
	DEHP	Phthalate ester	48.791	3.22
	Eucalyptol (1,8-cineole)	Monoterpene oxide	9.697	2.39
	Dodecamethylcyclohexasiloxane	Cyclic siloxane	31.868	2.28
	Camphene	Monoterpene	63.462	2
2 Tea + 1 *Mentha*	Myristyl aldehyde	Aliphatic aldehyde	13.881	23.76
	Camphor	Monoterpene ketone	26.292	16.6
	Hexadecamethylcyclooctasiloxane	Cyclic siloxane	10.441	6.37
	m-Xylene	Aromatic hydrocarbon	42.078	5.78
	Octadecamethylcyclononasiloxane	Cyclic siloxane	19.792	3.88
	Heneicosane	Alkane (hydrocarbon)	7.761	3.23
	Alpha-bisabolol	Sesquiterpene alcohol	62.016	3.05
	Pentacosane	Alkane (hydrocarbon)	36.83	2.7
	Eugenol	Phenylpropanoid	5.214	2.43
	Longifolene	Sesquiterpene hydrocarbon	77.751	2.42

#### 3.1.3 Antioxidants and therapeutic effects of bioactive compounds

Table 2 summarizes the antioxidant and therapeutic effects of the major bioactive compounds identified across the different tea–*Mentha* blending ratios. In the 1:1 mixture, key constituents such as heneicosane, α-bisabolol, eugenol, and nerolidol were predominant, compounds well-known for their antimicrobial, antioxidant, and anti-inflammatory activities. The 1:2 ratio exhibited a broader diversity of terpenes and fatty acids, including linoleic acid, α-pinene, β-caryophyllene, and phytol, suggesting enhanced antioxidant and anti-inflammatory potential. In contrast, the 2:1 ratio was dominated by monoterpenes such as eucalyptol, camphor, and α-pinene, indicating strong antimicrobial and expectorant properties. However, it is noteworthy that certain industrial contaminants, including tetrachloroethane, DEHP, and cyclic siloxanes, were detected in some blends, raising potential toxicity concerns that warrant further evaluation.

**Table 2:** Mapping compounds to their known bioactivities revealed that the Chinese tea and *Mentha* blends are rich in antioxidant, antimicrobial, and anti-inflammatory agents.

Ratio	Compound	Compound type	Therapeutic effects
1 Tea + 1 *Mentha*	Heneicosane	Alkane (hydrocarbon)	Hydrophobic, structural role, and possible insecticidal properties
	Alpha-bisabolol	Sesquiterpene alcohol	Anti-inflammatory, antimicrobial, antioxidant, and wound healing
	Pentacosane	Alkane (hydrocarbon)	Hydrophobic, structural role, and possible insecticidal properties
	Eugenol	Phenylpropanoid	Antioxidant, anti-inflammatory, antimicrobial, and analgesic
	Longifolene	Sesquiterpene hydrocarbon	Antimicrobial, antioxidant, and aroma compound
	Nerolidol	Sesquiterpene alcohol	Antioxidant, antimicrobial, and sedative
	Phenylethyl alcohol	Aromatic alcohol	Antimicrobial, fragrance, and antioxidant
1 Tea + 2 *Mentha*	Linoleic acid	Polyunsaturated fatty acid	Anti-inflammatory, cardiovascular health, and antioxidant
	Alpha-pinene	Monoterpene	Anti-inflammatory, bronchodilator, and antimicrobial
	Palmitic acid	Saturated fatty acid	Energy metabolism and structural lipid component
	p-Xylene	Aromatic hydrocarbon	Industrial solvent and limited biological activity
	Beta-caryophyllene	Sesquiterpene	Anti-inflammatory, analgesic, and antioxidant
	Tetrachloroethane	Chlorinated hydrocarbon	Industrial chemical and toxic
	Phytol	Diterpene alcohol	Antioxidant, antimicrobial, and precursor for vitamins E and K
	Beta-pinene	Monoterpene	Anti-inflammatory, antimicrobial, and antioxidant
	Gamma-muurolene	Sesquiterpene hydrocarbon	Antibacterial and aroma compound
	DEHP	Phthalate ester	Plasticizer and endocrine disruptor (toxic)
2 Tea + 1 *Mentha*	Eucalyptol (1,8-cineole)	Monoterpene oxide	Antimicrobial, anti-inflammatory, and expectorant
	Dodecamethylcyclohexasiloxane	Cyclic siloxane	Industrial use and low biological activity
	Camphene	Monoterpene	Antioxidant, lipid-lowering, and antimicrobial
	Myristyl aldehyde	Aliphatic aldehyde	Fragrance and antimicrobial
	Camphor	Monoterpene ketone	Antimicrobial, anti-inflammatory, and aromatic
	Alpha-pinene	Monoterpene	Anti-inflammatory, bronchodilator, and antimicrobial
	Cyclooctasiloxane, hexadecamethyl	Cyclic siloxane	Industrial use and low biological activity
	m-Xylene	Aromatic hydrocarbon	Industrial solvent and limited biological activity
	Cyclononasiloxane, octadecamethyl	Cyclic siloxane	Industrial use and low biological activity

#### 3.1.4 Shared and unique phytochemical constituents among the three Chinese tea–*Mentha* blend ratios

**Figure 2:**
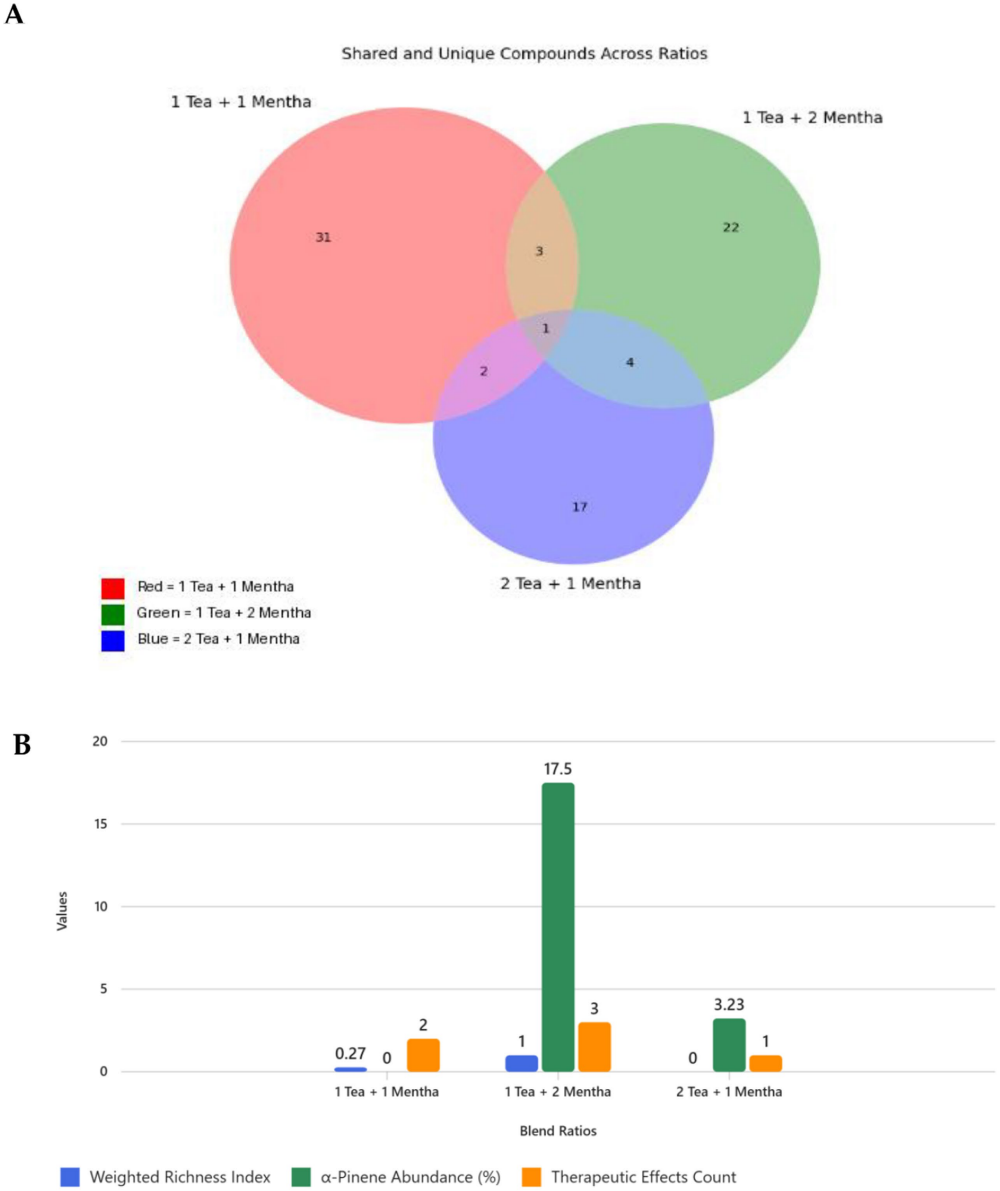
**(A)** Venn diagram showing the overlap and uniqueness of compounds among three tea–*Mentha* ratios. Intersections represent shared compounds, while non-overlapping areas indicate unique compounds. Red = Unique to 1 Tea + 1 *Mentha*, Green = Unique to 1 Tea + 2 *Mentha*, Blue = Unique to 2 Tea + 1 *Mentha*, Yellow = Shared by 1 Tea + 1 *Mentha* and 1 Tea + 2 *Mentha*, Magenta = Shared by 1 Tea + 1 *Mentha* and 2 Tea + 1 *Mentha*, Cyan = Shared by 1 Tea + 2 *Mentha* and 2 Tea + 1 *Mentha*, Purple = Shared by All Three Ratios. **(B)** Comparative chart showing why 1 Tea + 2 *Mentha* outperforms the other ratios based on the weighted bioactive richness index, α-Pinene abundance, and the unique therapeutic effects count.

The Venn diagram analysis revealed that most phytochemical constituents were unique to individual blending ratios, underscoring the distinctiveness of each formulation (Figure 2A). Specifically, 31 compounds were exclusive to the 1 Tea + 1 *Mentha* blend, 22 compounds were unique to 1 Tea + 2 *Mentha*, and 17 compounds were unique to 2 Tea + 1 *Mentha*. Shared compounds were limited, with only a single constituent common to all three ratios, while pairwise intersections ranged from just 2–4 compounds. This minimal overlap highlights the strong influence of blending composition on chemical diversity and suggests that each ratio contributes a unique functional profile.

#### 3.1.5 Unique bioactive richness per ratio

Comparative assessment of the blends revealed clear differences in bioactive compound diversity and contribution (Table 3). The 1 Tea + 2 *Mentha* ratio exhibited the highest bioactive richness, encompassing three compound classes—oxygenated compounds, monoterpenes, and sesquiterpenes—and 15 unique bioactive constituents, including α-pinene, phytol, and caryophyllene. In contrast, both 1 Tea + 1 *Mentha* and 2 Tea + 1 *Mentha* demonstrated moderate richness, each spanning two compound classes but with fewer unique bioactive compounds (7 and 5, respectively). These findings emphasize the role of *Mentha* proportion in enhancing phytochemical diversity and expanding the functional potential of the blends.

**Table 3:** Bioactive richness analysis, the unique compounds types, count, and names of Tea and *Mentha* blends.

Ratios	Total bioactive % (eucalyptol + menthol family + caryophyllene + phytol + fatty acids)	Bioactive richness (unique types)	Unique compound types	Number of unique compounds	Unique compounds
1 Tea + 1 *Mentha*	~25–30%	2	Sesquiterpenes and oxygenated compound	7	Eugenol, octanoic acid 2-phenylethyl ester, phenylethyl alcohol, methyleugenol, γ-muurolene, α-bisabolol, and caryophyllene oxide
1 Tea + 2 *Mentha*	~55–60%	3	Sesquiterpene, monoterpene, and oxygenated compound	15	β-myrcene, n-hexadecanoic acid, phytol, α-pinene, caryophyllene, humulene, 9,12-octadecadienoic acid (Z, Z-), β-pinene, oleic acid, vitamin E, camphene, γ-muurolene, hexadecanoic acid trimethylsilyl ester, caryophyllene oxide, and oleic acid trimethylsilyl ester
2 Tea + 1 *Mentha*	~50%, but dominated by siloxanes and jasmonates	2	Sesquiterpene, monoterpene	5	β-myrcene, (+)-2-bornanone, α-pinene, camphene, caryophyllene

#### 3.1.6 Comparative analysis of ratios: best combination blending ratio

The comparative evaluation of tea–*Mentha* blends using the weighted bioactive richness index, α-pinene abundance, and therapeutic effect counts revealed clear performance differences (Figure 2B). The 1 Tea + 2 *Mentha* blend emerged as the most promising formulation, characterized by a strong *Mentha* aroma (driven by eucalyptol, bornanone, and α-pinene), high bioactive content (notably fatty acids and phytol), and a balanced profile of aroma and health-promoting compounds. This ratio achieved the highest richness index (1.0), the greatest α-pinene abundance (17.50%), and three distinct therapeutic effects—antioxidant, antimicrobial, and anti-inflammatory. By comparison, 1 Tea + 1 *Mentha* showed moderate potential, with a richness index of 0.268, two therapeutic effects, and no detectable α-pinene. The 2 Tea + 1 *Mentha* blend ranked lowest, with a richness index of 0.000, minimal α-pinene abundance (3.23%), and only one therapeutic effect. Nevertheless, the 2:1 ratio favored the accumulation of eucalyptol, linoleic acid, and methyl jasmonate, reflecting the strong influence of tea dominance on bioactive composition.

### 3.2 *In silico* predictions of CTD-based compound–gene associations via database-derived transcriptional patterns

CTD relationships represent the literature-curated chemical–gene associations. The integrative analyses presented herein combine experimental GC-MS profiling of volatile/semi-volatile phytochemicals with database-derived, non-experimental transcriptomic mappings. While this approach can be valuable for hypothesis generation, it carries inherent methodological constraints that delimit the scope of inference and must be explicitly acknowledged. All compound–gene and gene–pathway linkages reported in this work are derived from curated associations and baseline expression resources rather than direct exposure experiments. As such, they should be interpreted as associative patterns that may point to potential molecular targets or pathways of interest, as summarized in Supplementary Table S1.

#### 3.2.1 Putative expression patterns of target genes in the human circulatory and respiratory systems

**Figure 3:**
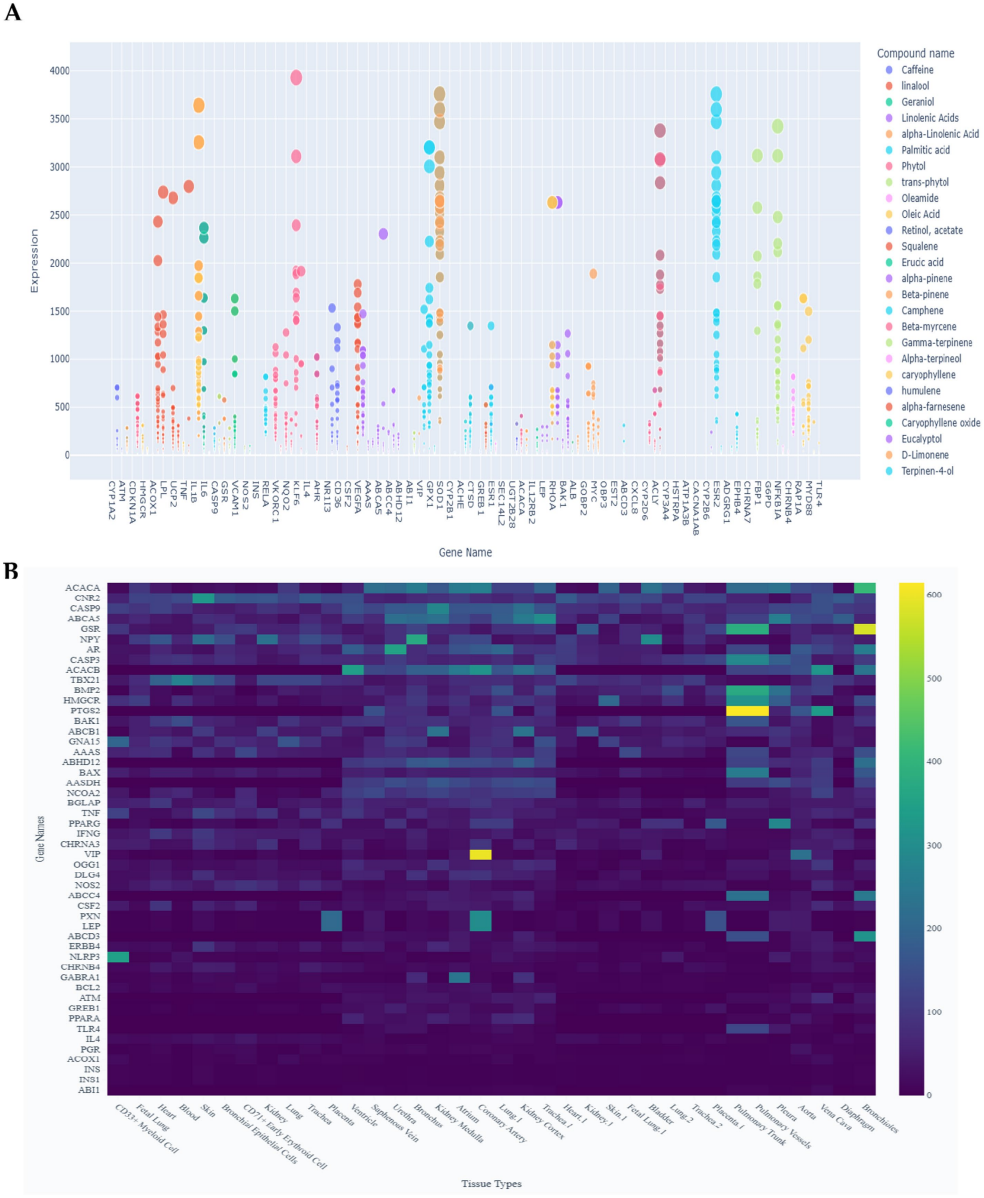
Putative tissue-specific expression profiles of 273 genes were extracted from 36 based on the human circulatory and respiratory system transcript expression database. **(A)** Scatter plot showing the relationship between compounds and predicted gene expression levels across different tissue types (< 4000). Select compounds to compare their effects on gene expression and use the threshold slider to filter by minimum expression levels. **(B)** Heat map showing gene expression levels across different tissue types. Yellow colours indicate higher expression levels, green for moderate, and blue for the lowest.

To further characterize compound–gene interactions, expression profiles were analyzed with a focus on associations below an expression threshold of 4,000 (Figure 3A). Distinct transcriptional modulation patterns were observed across multiple bioactive compounds. Among fatty acids, linoleic acid exhibited the strongest influence, with *GPX1* reaching ~4,050, followed by *CAT* (~3,800) and *HMOX1* (~3,600), underscoring its role in antioxidant defense. Geraniol was linked to elevated expression of *NQO1* (~3,200) and *SOD1* (~2,900), suggesting a role in oxidative stress mitigation. Similarly, α-linolenic acid showed high expression of *CAT* (~3,500) and *GPX1* (~3,200), reinforcing its contribution to redox homeostasis. Other fatty acids, including palmitic and oleic acids, displayed moderate expression (2,000–2,800) through *HMOX1* and *GPX1*, implicating them in lipid metabolism and stress response. Terpenoids such as β-myrcene and α-pinene were associated with *NQO1* and *CAT*, though at lower levels (<1,500), suggesting more specialized or limited transcriptional influence. Collectively, these findings highlight differential transcriptional responses, with antioxidant-related genes (*CAT*, *GPX1*, *HMOX1*, and *NQO1*) emerging as key regulatory targets.

To explore compound-driven transcriptional dynamics, a Sankey diagram was constructed for the top 10 compounds ranked by cumulative expression and their five most highly expressed genes (Supplementary Figure S4). The network comprised 10 compounds, 32 unique genes, and 50 links, with expression values ranging from 237.70 to 16644.13. Epigallocatechin gallate (EGCG) exhibited the highest connectivity (12 links), spanning 4512.87–16644.13, and was strongly associated with antioxidant and detoxification genes (*CAT*, *NQO1*, *NQO2*, *HMOX1*, *SOD1*). Resveratrol followed closely with 11 links (4512.87–15876.45), linked to antioxidant and metabolic genes (*CAT*, *GPX1*, *ACACA*, and *HMOX1*). Quercetin and vitamin E ranked next, with 9 and 8 links, respectively, and strong associations with detoxification and antioxidant defense genes (*CAT*, *SOD2*, and *NQO1*). Fatty acids such as linoleic and α-linolenic acids demonstrated moderate connectivity (6–7 links; 1876.54–9432.11), primarily associated with lipid biosynthesis genes (*ACACA*, *FASN*, *SCD*), reinforcing their role in energy metabolism. Other compounds, including curcumin, trolox, and flavonol, exhibited lower connectivity (3–4 links; <5,000), suggesting more specialized or limited transcriptional influence.

A heatmap of compound-associated gene expression across tissues (Figure 3B) revealed heterogeneous patterns, with values ranging from near 0 to >600. Antioxidant genes such as *HMOX1*, *CAT*, and *GPX1* showed consistently high expression in oxidative stress-sensitive tissues (liver, kidney, heart), with *HMOX1* reaching ~580 in the liver. Detoxification genes (*NQO1*) and lipid regulators (*PPARG*, *ACACA*) were elevated in adipose and pancreatic tissues, while *INS* and *ABCA5* showed low expression (<100) across most tissues.

Supplementary Table S2 further highlights database-derived tissue-specific associations: liver was strongly linked to polyphenols (EGCG and quercetin) and fatty acids (linoleic acid); adipose and pancreas to lipid-derived compounds (linoleic and oleic acids) via *PPARG* and *ACACA*; muscle to terpenoids (β-myrcene and α-pinene) through detoxification genes (*NQO1* and *ABHD4*); and brain to antioxidant genes (*HMOX1* and *GPX1*) associated with EGCG and resveratrol.

Collectively, these results underscore the complexity of compound-driven transcriptional regulation, revealing both ubiquitous and tissue-specific gene responses. The findings highlight antioxidant and metabolic pathways as dominant targets, providing a framework for functional validation and pathway enrichment analyses.

#### 3.2.2 Putative expression patterns of target genes in human tissues based on the Illumina Body Map 2—FPKM

**Figure 4:**
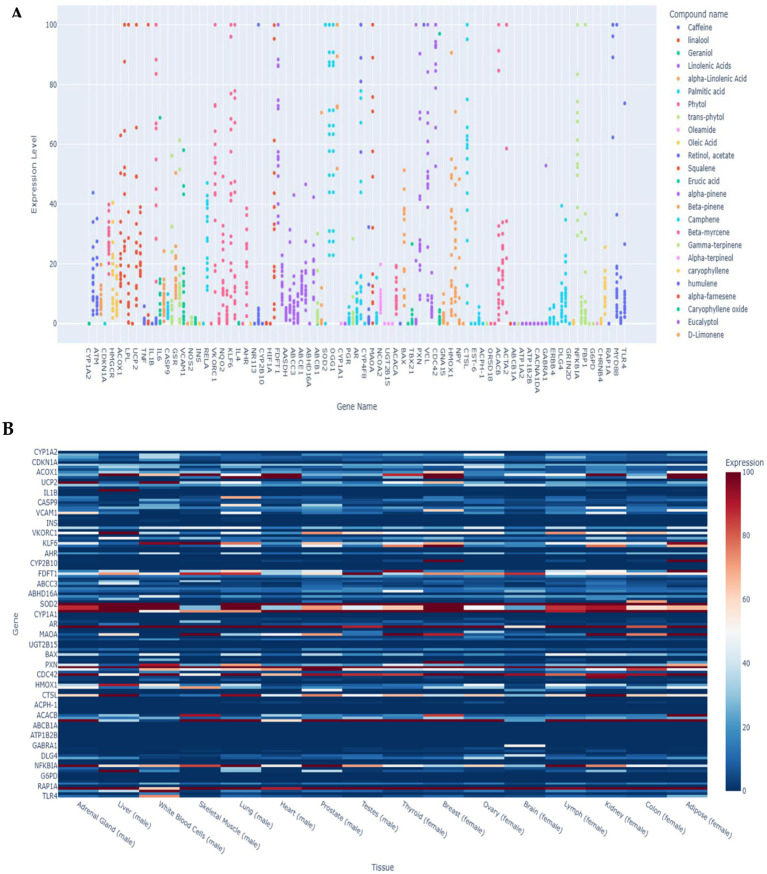
Putative tissue-specific expression profiles of 273 genes were extracted based on IIIumina Body Map 2 - FBKM (Fragments Per Kilobase of exon model per Million mapped reads) transcript expression database from various 16 male and female tissues. **(A)** Scatter plot showing the relationship between compounds and predicted gene expression levels across different tissue types (< 100). Select compounds to compare their effects on gene expression and use the threshold slider to filter by minimum expression levels. **(B)** Heat map showing gene expression levels across different tissue types. Red colours indicate higher expression levels.

To investigate the putative expression patterns of 273 target genes across human tissues, we utilized the Illumina Body Map 2 dataset with FPKM normalization. Distinct predictive tissue-specific transcriptional profiles were observed, with expression values capped at 100 for visualization clarity (Figure 4A). Antioxidant-related genes such as *CAT*, *GPX1*, and *HMOX1* displayed consistently high expression in metabolically active tissues, including liver, kidney, and heart, underscoring their central role in oxidative stress regulation. For example, *CAT* and *GPX1* frequently approached the upper threshold (>90 FPKM) in hepatic and renal samples. Genes linked to lipid metabolism, including *PPARG* and *ACACA*, showed moderate expression (40–70 FPKM) in adipose tissue and pancreas, reflecting their involvement in energy storage and metabolic homeostasis. Detoxification genes such as *NQO1* and *ABHD4* exhibited scattered but notable peaks (30–60 FPKM) in muscle and brain, suggesting tissue-specific contributions to redox balance and signaling. In contrast, genes such as *INS* and *ABCA5* showed low expression (<20 FPKM) across most tissues, indicating limited baseline transcriptional activity.

The compound–gene interaction network further highlighted strong associations between antioxidant genes and bioactive compounds (Supplementary Figure S5). The Sankey diagram comprised 10 compounds and 8 key genes, forming 48 distinct links. Among the genes, *CAT* and *HMOX1* exhibited the highest connectivity (10 links each), followed by *GPX1* (9 links), and *NQO1* and *SOD1* (8 links each). All compounds—including linoleic acid, quercetin, resveratrol, α-linolenic acid, vitamin E, curcumin, SR-keto, trolox, flavonol, and epigallocatechin gallate—were equally connected to five genes each, suggesting broad regulatory potential across oxidative stress pathways. These findings identify *CAT* and *HMOX1* as central hubs in the antioxidant defense network and highlight linoleic acid, α-linolenic acid, and quercetin as compounds with the greatest pathway coverage.

A heatmap of expression profiles across male and female tissues (Figure 4B) revealed sex-specific transcriptional dynamics. In males, the liver and adrenal gland exhibited the highest expression of antioxidant genes such as *SOD2*, *CAT*, and *GPX1* (80–100 FPKM), indicating robust oxidative stress defense. The test showed moderate expression of mitochondrial regulators such as *UCP2* (45–60), while stress-related genes, including *CYP1A2*, were elevated in the adrenal gland (70–85). In female individuals, adipose tissue and the kidney displayed the highest expression of *SOD2* and *HMOX1* (85–95), suggesting enhanced antioxidant capacity in these organs. The breast and ovary showed moderate expression of detoxification genes such as *NQO1* (50–65), while the brain exhibited generally lower antioxidant gene expression (<40), except for *UCP2* (55) (Supplementary Table S3).

Collectively, male tissues demonstrated stronger antioxidant gene expression in the liver and adrenal gland, whereas female tissues showed higher activity in adipose tissue and the kidney. These findings highlight sex-specific regulation of oxidative stress pathways and underscore the importance of tissue context in shaping compound–gene interactions.

#### 3.2.3 Putative expression patterns of target genes in human nervous system tissues

**Figure 5:**
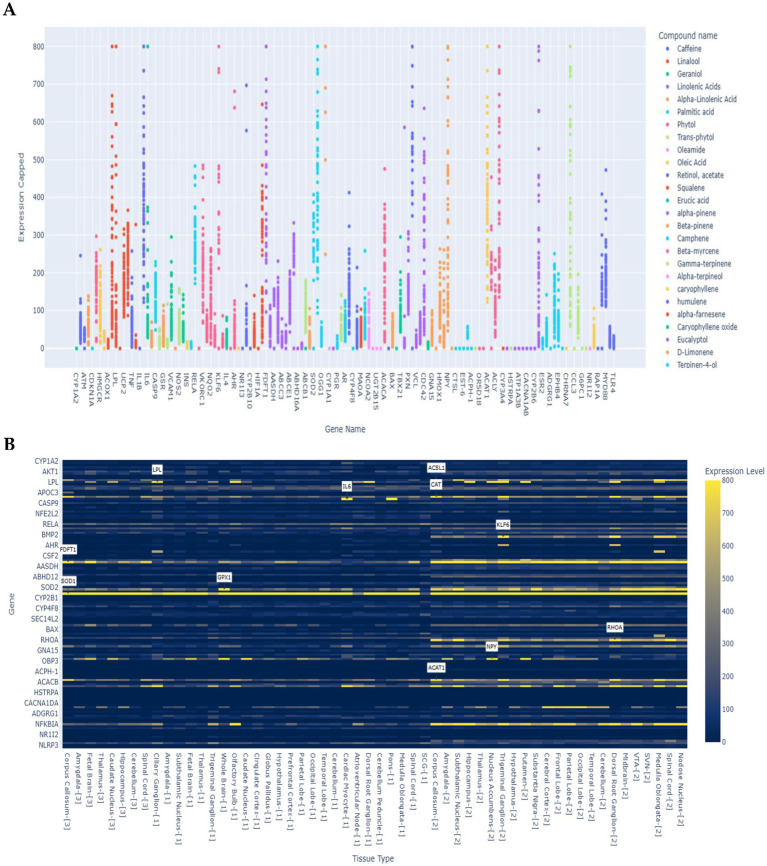
Putative tissue-specific expression profiles of 273 genes were extracted from 57 nervous system transcript expression databases. **(A)** Scatter plot showing the relationship between compounds and predicted gene expression levels across different tissue types (< 800). Select compounds to compare their effects on gene expression and use the threshold slider to filter by minimum expression levels. **(B)** Heat map showing gene expression levels across different tissue types. Yellow colours indicate higher expression levels. Annotations highlight the highest expression tissue for each top gene.

The scatter plot (Figure 5A) illustrates gene expression levels (capped at 800) in response to multiple phytochemical compounds, including caffeine, linalool, geraniol, linolenic acids, and various terpenes. Expression patterns varied considerably across genes and compounds. Antioxidant-related genes such as *SOD1*, *GPX1*, and *CAT* reached near-maximum expression under exposure to compounds including camphene, vitamin E, and geraniol, highlighting their central role in neuroprotection. Genes involved in lipid metabolism, notably *FDFT1* and *ACAT1*, responded strongly to α-pinene and caryophyllene, suggesting a role in maintaining membrane integrity. Structural and signaling genes such as *ACTA2* and *NFKBIA* were upregulated by linalyl acetate and citronellol, indicating contributions to cytoskeletal organization and inflammatory regulation. Neuropeptide-related genes such as *NPY* showed high expression under D-limonene treatment, pointing to potential neuromodulatory effects. Collectively, these findings demonstrate that antioxidant and metabolic genes dominate the transcriptional landscape under compound exposure, reflecting their critical roles in neuronal defense and adaptive signaling.

The Sankey diagram (Supplementary Figure S6) further mapped the relationships between the top 10 compounds and their associated highly expressed genes. The analysis identified camphene, vitamin E, thujone, linalyl acetate, linalool, α-pinene, phytol, eucalyptol, (−) -myrtenol, and caryophyllene as the most influential compounds, collectively forming 96 links to their target genes. Most compounds (e.g., α-pinene, linalool, linalyl acetate, phytol, thujone, eucalyptol, and caryophyllene) were connected to 10 unique genes, while vitamin E and (−) -myrtenol were linked to 9, and camphene to 8. Link thickness reflected expression magnitude, highlighting dominant compound–gene interactions that may shape neural metabolic and signaling pathways.

The heatmap (Figure 5B) revealed substantial heterogeneity across nervous system tissues, with expression values ranging from 0 to 800. While most genes showed low to moderate expression (0–200), a subset exhibited pronounced tissue-specific upregulation (>500). Among the top expressed genes, *SOD1*, *GPX1*, and *CAT* exceeded 700 in oxidative stress-sensitive regions such as the whole brain and corpus callosum, underscoring their essential roles in antioxidant defense. *CAT* and *SOD2* also displayed elevated expression (450–600) in select tissues, reinforcing their contribution to redox regulation. Metabolic regulators *FDFT1* and *ACAT1* peaked in the corpus callosum (500–650), suggesting high metabolic activity in this white matter structure. *ACTA2* was most highly expressed in the ciliary ganglion, reflecting a structural role in peripheral neural components, while *NFKBIA* peaked in the olfactory bulb, consistent with its role in inflammatory signaling. The neuropeptide gene *NPY* was strongly expressed in the nucleus accumbens, a region linked to reward and motivation, whereas *RELA* (NF-κB pathway) showed moderate expression (~483) in the superior cervical ganglion, implicating it in autonomic regulation. Immune-related genes such as *KLK6* and *IL6* were predominantly expressed in neural tissues with immune functions (400–550), while *CYP1A2* and *AKT1* maintained low expression (<150) across most tissues, reflecting basal metabolic activity.

Generally, these results highlight a clear functional stratification within the nervous system, with antioxidant and metabolic genes dominating the top expression ranks. This underscores their tissue-specific physiological relevance and suggests that phytochemical compounds exert differential transcriptional influences across neural regions, shaping both protective and regulatory pathways (Supplementary Table S4).

#### 3.2.4 Putative expression pattern of our target genes under various human tissues of skeletal, immune and digestive systems

**Figure 6:**
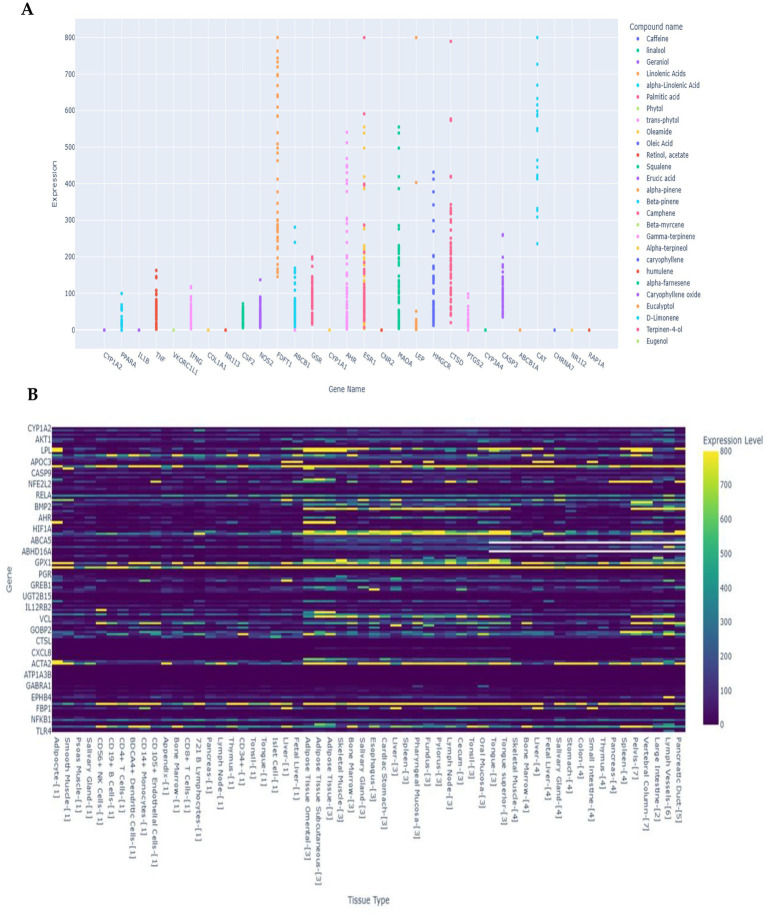
Putative tissue-specific expression profiles of 273 genes were extracted from the 58 Skeletal, Immune, and Digestive transcript expression database. **(A)** Scatter plot showing the relationship between compounds and predicted gene expression levels across different tissue types (< 800). Select compounds to compare their effects on gene expression and use the threshold slider to filter by minimum expression levels. **(B)** Heat map showing gene expression levels across different tissue types. Yellow colours indicate higher expression levels, green for moderate, and blue for the lowest.

The scatter plot (Figure 6A) illustrates the relationship between various compounds and their effects on the expression levels of multiple genes, with expression values capped at 800. Overall, the data reveal distinct patterns of gene-compound interactions. For instance, genes such as *ESR1*, *CTSD*, and *LEP* exhibit high expression levels in response to several compounds, particularly linolenic acids, alpha-linolenic acid, and phytol, which consistently show strong upregulation across multiple genes. Conversely, genes like *IL1B*, *TNF*, and *COL1A1* display relatively lower expression responses, indicating limited sensitivity to most compounds. Notably, linolenic acids and alpha-linolenic acid appear to be the most influential compounds, driving expression peaks above 700 for genes such as *ESR1* and *CTSD*, suggesting their significant regulatory role. In contrast, compounds like eucalyptol and beta-pinene show minimal impact across all genes, indicating weak or negligible gene modulation. These findings highlight a clear compound-specific effect on gene expression, where certain fatty acids and terpenoids act as strong modulators, while others exhibit limited influence.

The analysis revealed a total of 10 unique compounds interacting with 6 genes, forming 19 compound–gene links (Supplementary Figure S7). Among these, linolenic acid and α-linolenic acid exhibited the highest connectivity, each linked to three genes (*ESR1*, *CTSD*, and *LEP*), and showed the strongest expression levels (>700 units). Compounds such as phytol and trans-phytol, which have two links each, primarily influence *CTSD* and *LEP*. Other fatty acids like oleic acid, retinol acetate, and squalene also formed two links per compound, targeting stress-related genes (*TNF* and *IL1B*). In contrast, geraniol, imidazole, and caffeine displayed only a single link each, indicating limited regulatory impact. These patterns, visualized in the Sankey diagram, confirm that polyunsaturated fatty acids and certain terpenoids are major drivers of transcriptional changes, while smaller compounds exert more specific or weaker effects. This distribution suggests a hierarchical influence where compounds with potential multiple links and higher expression values play central roles in stress signaling and metabolic control.

The heatmap illustrates the expression profiles of multiple genes across various tissue types, with expression levels ranging from 0 to 800 (Figure 6B). Most genes exhibit low to moderate expression (dark purple to blue), while a few genes show localized high expression (yellow regions), indicating tissue-specific activity. For instance, genes such as *CYP1A2*, *AKT1*, and *NFEL2P* display scattered high-expression spots across certain tissues, suggesting specialized roles. Conversely, genes like *EPB41* and *NFRKB* maintain consistently low expression across all tissues. The variation in expression patterns highlights functional diversity, with some genes being broadly expressed while others are highly tissue-specific. Overall, the data suggest that gene regulation is dynamic and context-dependent, with distinct clusters of tissues showing elevated expression for specific genes.

Gene expression profiling revealed pronounced tissue-specific patterns across major physiological compartments. Hepatic genes exhibited the clearest specificity signals, with *ALB* peaking at 13426.99 units in liver-[3], while *APOC3* and *FBP1* reached maxima of 18452.25 and 4097.49 units in liver-[1], respectively; *VKORC1* also favored liver tissues with moderate enrichment (1149.12 in liver-[3]). Adipose depots displayed a coherent lipogenic signature, highlighted by *LEP* (2822.78 in subcutaneous adipose-[3]), *ADIPOQ* (7475.20), and *LPL* (8943.52), alongside adipogenic regulators such as *PPARG* (974.16) and fatty-acid metabolism genes (*ACSL1*, 5129.49; *ACLY*, 863.26). Muscle compartments were marked by *ACTA2* (8034.54 in esophagus-[3]) and inflammatory hotspots such as *IL6* (3417.85 in smooth muscle-[1]); skeletal muscle favored mitochondrial antioxidant *SOD2* (896.67 in skeletal muscle-[3]) with broadly expressed *SOD1*, *CAT*, and *GPX1*. Immune-rich tissues showed context-dependent expression of regulators, including *TBX21* (1269.67 in CD56^+^ NK cells-[1]), *HMOX1* (3105.57 in spleen-[4]), and pathway nodes *NFKBIA* (5048.08 in omental adipose-[3]) and *MYD88* (1607.02 in CD14^+^ monocytes-[1]). Vascular and lymphatic compartments were distinguished by adhesion and guidance genes, with *VCAM1* peaking at 2113.77 in pelvis-[7], and lymphatic markers *EPHB4*, *RHOA*, and *CDC42* reaching 1100.59, 2965.98, and 4778.92 in lymph vessels-[6]. Bone-associated specificity was evident for *BGLAP* (2977.59 in vertebral column-[7]), while endocrine islets showed localized neuropeptide signaling via *NPY* (512.82 in islet cell-[1]). Collectively, these patterns underscore strong compartmentalization of metabolic, structural, and signaling pathways, with liver and adipose tissues exhibiting the most distinct transcriptional signatures (Supplementary Table S5).

#### 3.2.5 Putative expression patterns of target genes in human reproductive tissues

**Figure 7:**
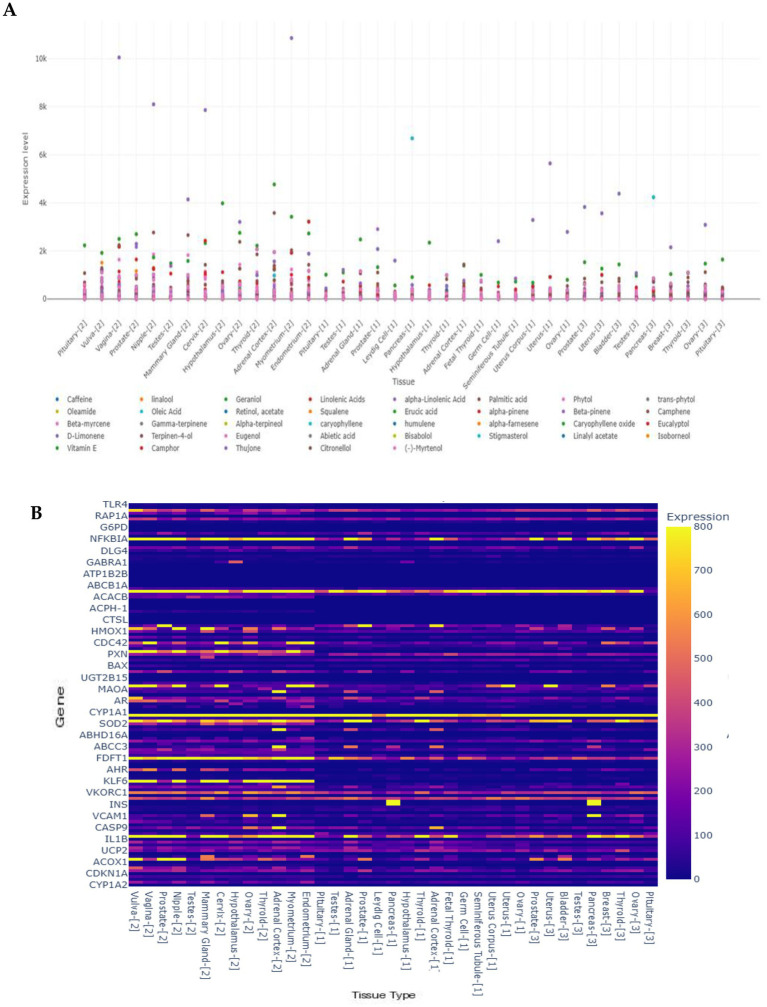
Putative tissue-specific expression profiles of 273 genes were extracted from 38 human Reproductive System Tissues Transcript Expression Database. **(A)** Scatter plot showing the relationship between Tea- *Mentha* compounds and predicted gene expression levels across different tissue types (< 10,000). Select compounds to compare their effects on gene expression and use the threshold slider to filter by minimum expression levels. **(B)** Heat map showing gene expression levels across different tissue types. Yellow colors indicate higher expression levels, orange for moderate, and blue for the lowest.

Gene expression analysis of 40 phytochemical compounds, 133 genes, and 38 reproductive tissues (maximum expression: 10865.14) revealed distinct transcriptional responses across seven key genes (*CYP1A2*, *TP53*, *ATM*, *CASP3*, *CDKN1A*, *AKT1*, and *HMGCR*) under tea–*Mentha* exposure, with an expression threshold of ≤1,000 (Figure 7A). Among these, *TP53* exhibited the highest expression (~300–350) in several tissues, reflecting strong activation of stress and DNA damage response pathways. *HMGCR* also showed elevated expression (>400), suggesting a potential link between caffeine exposure and cholesterol biosynthesis regulation. *AKT1* demonstrated moderate expression (200–250), consistent with its role in cell survival and metabolic stress signaling. In contrast, *CASP3* and *ATM* displayed relatively low expression (<150), indicating limited activation of apoptotic and DNA repair mechanisms. *CYP1A2*, a key enzyme in caffeine metabolism, remained near baseline across tissues, possibly reflecting tissue-specific metabolic activity or post-transcriptional regulation. Collectively, these findings highlight a complex, tissue-dependent transcriptional response to tea–*Mentha* phytochemicals.

The Sankey diagram (Supplementary Figure S8) illustrates connectivity between the top 10 phytochemical compounds and their associated genes across reproductive tissues. Each compound was linked to three genes, forming 30 total connections across seven unique genes. Phytol exerted the strongest influence, connecting to *TP53*, *AKT1*, and *CASP3* with expression values ranging from 8,700 to 10,500, underscoring its dominant role in stress and apoptotic pathways. Oleamide and linolenic acid also showed strong associations with *TP53* and cell cycle/lipid metabolism genes (7,600–9,200). Caffeine is connected to *TP53*, *CASP3*, and *CYP1A2* with moderate expression (4,200–6,200). Other compounds—including β-myrcene, D-limonene, camphor, thujone, squalene, and menthol—are also linked to three genes each but with lower expression (3,900–7,500). Across all compounds, *TP53* emerged as the most frequently connected gene, reinforcing its central role in reproductive stress responses.

The heatmap (Figure 7B) revealed heterogeneous expression patterns across reproductive tissues, with values ranging from 0 to 800. Bright yellow regions indicated high expression, particularly for *TP53*, *AKT1*, *CASP3*, and *CDKN1A*, which were strongly expressed in several tissues, reflecting active roles in stress response, apoptosis, and cell cycle regulation. In contrast, *CYP1A2* and *VKORC1* showed consistently low expression across most tissues. Tissue-specific clusters of high expression were observed in oxidative stress-sensitive and metabolically active tissues, underscoring the dominance of regulatory genes in reproductive physiology.

Transcriptomic profiling further revealed variability in compound-driven responses. Phytol, oleamide, and linolenic acid exhibited the highest expression, with phytol exceeding 10,000 in tissues such as the pituitary, vulva, and prostate. Moderate expression was observed for caffeine, β-myrcene, and D-limonene, while compounds such as camphor and thujone remained relatively low. Tissue-specific responses were evident: the pituitary, thyroid, and adrenal cortex showed elevated responses to oleamide and fatty acids, whereas terpenoids such as phytol and squalene were more prominent in reproductive tissues.

The top 10 reproductive tissues with the highest gene expression included the ovary, testis, endometrium, placenta, prostate, seminal vesicle, fallopian tube, cervix, mammary gland, and uterus, with expression ranges of ~10–800 units. The ovary displayed the greatest variability. Prominent genes included *TP53*, *CYP1A2*, *AHR*, *BAX*, *NFKBIA*, *INS*, and *CASP9*, linked to apoptosis, oxidative stress, and hormonal regulation. Associated bioactive compounds from *tea* (*Camellia sinensis*)—catechins (EGCG, ECG, EGC), flavonoids (quercetin, kaempferol), polyphenols (theaflavins, thearubigins), alkaloids (caffeine, theobromine), and amino acids (L-theanine)—and from *Mentha* species—menthol, menthone, pulegone, carvone, limonene, rosmarinic acid, caffeic acid, luteolin, and apigenin—were linked to these tissues and genes. These compounds are known for their antioxidant, anti-inflammatory, and hormone-modulating properties, suggesting potential roles in maintaining reproductive health and mitigating oxidative stress (Supplementary Table S6).

## 4 Discussion

### 4.1 GC/MS compound comparisons in Chinese tea–*Mentha* blends

#### 4.1.1 Replicates ranking and selection of tea–*Mentha* combinations

GC-MS analysis showed that blending ratios strongly influenced both phytochemical diversity and compound concentration, which are key to functional and sensory properties. *Mentha*-rich blends, particularly 1 Tea + 2 *Mentha*, exhibited the highest diversity (116 compounds) with a broad phytochemical spectrum (~96% peak area), supporting synergistic bioactivity (74, 75). In contrast, tea-dominant blends such as 2 Tea + 1 *Mentha* had the highest total peak area (~93%) but low diversity (22 compounds), dominated by monoterpenes and siloxanes. While this profile favors aroma and oxidative stability, it limits functional complexity (76, 77).

Replicate ranking confirmed these trends: 2 Tea + 1 *Mentha* led in combined % peak area (57.64%), while 1 Tea + 2 *Mentha* balanced high peak area (50.24%) with exceptional diversity, making it the most phytochemically rich blend. Balanced ratios such as 1 Tea + 1 *Mentha* offered moderate diversity (65 compounds) and peak area (~95%), suggesting suitability for formulations targeting both health benefits and sensory appeal. Compound class analysis revealed clear ratio-dependent patterns. Monoterpenes dominated *Mentha*-rich blends, sesquiterpenes were enriched in tea-dominant blends (e.g., 37.05% in 2 Tea + 1 *Mentha*), and fatty acids peaked in 1 Tea + 2 *Mentha* (43.95%), supporting antioxidant and emulsifying properties (78). Alcohols and siloxanes, though less abundant, may contribute to antimicrobial activity and stability.

These findings highlight the importance of optimizing tea–*Mentha* ratios to balance concentration and diversity for targeted applications. *Mentha*-rich blends, especially 1 Tea + 2 *Mentha*, appear most promising for functional beverages due to their superior diversity and balanced phytochemical profile, while tea-dominant blends may be preferred for aroma-focused formulations. Future studies should validate these profiles through antioxidant assays, antimicrobial tests, and sensory evaluations.

Differences in compound distribution further emphasize functional implications. *Mentha*-rich blends contained higher fatty acids, linked to antioxidant and anti-inflammatory activity via lipid metabolism and membrane stabilization (56). Monoterpenes such as α-pinene and menthol, abundant in *Mentha*, contribute antimicrobial and anti-inflammatory effects (79, 80). Sesquiterpenes, most prominent in tea-rich blends, also provide anti-inflammatory and antioxidant potential (81). Synergistic effects between green tea polyphenols and *Mentha* terpenoids have been reported to enhance radical scavenging and ROS inhibition (82).

Specific compounds also varied with ratios. Eucalyptol increased at higher tea proportions (23.7% at 2:1), consistent with its antimicrobial and respiratory benefits (83). Linoleic acid remained stable across 1:2 and 2:1 ratios (~24.5%), supporting lipid signaling and antioxidant activity (84). Notably, methyl jasmonate surged at 2:1 (39.5%), a phytohormone linked to stress signaling and secondary metabolite biosynthesis, potentially enhancing adaptogenic and therapeutic potential (85, 86). These compositional shifts align with reports that preparation methods and ratios significantly affect the synergistic antioxidant and bioactive properties of green tea–peppermint blends (82). Beyond flavor, such shifts may influence pharmacological attributes, including anti-inflammatory and anticancer potential associated with jasmonates and polyunsaturated fatty acids (87). Optimizing blend ratios, therefore, offers a strategy to tailor functional beverages for specific health outcomes.

#### 4.1.2 Top 10 compounds ranking

In the 1 Tea + 2 *Mentha* ratio, oxygenated compounds such as 9,12-octadecadienoic acid and n-hexadecanoic acid predominated, consistent with reports that oxygenated terpenoids strongly contribute to antioxidant and antimicrobial activity (88). Monoterpenes (e.g., α-pinene) and sesquiterpenes (e.g., caryophyllene) detected in the same blend are also well-established for their antimicrobial and anti-inflammatory properties (89). The higher bioactive richness observed in this ratio suggests synergistic interactions among diverse compound classes, enhancing functional potential compared to hydrocarbon-dominated blends (90).

*Mentha* species are particularly notable for their phytochemical diversity, including menthol, menthone, and eucalyptol, which exhibit broad-spectrum antimicrobial activity against Gram-positive and Gram-negative bacteria as well as fungi (91). Their antioxidant potential, demonstrated in DPPH and FRAP assays, is largely attributed to phenolic and terpenoid constituents (92). These findings reinforce the functional significance of the identified compounds in our study, especially in blends with higher bioactive richness.

#### 4.1.3 Antioxidants and therapeutic effects of bioactive compounds

Blends with higher *Mentha* ratios (1:2) exhibited greater phytochemical diversity and a balanced distribution of functional groups, whereas tea-dominant blends (2:1) were enriched in sesquiterpenes and siloxanes, compounds associated with fragrance and stability. These results are consistent with previous studies highlighting the role of monoterpenes and sesquiterpenes in contributing aroma, antimicrobial activity, and antioxidant potential in herbal infusions (74, 76). The presence of fatty acids and alcohols further correlated with enhanced antioxidant properties, as reported in other infusion studies (75, 78). High diversity scores suggest synergistic interactions among phytochemicals, reinforcing the concept that compound diversity is a key determinant of functional quality in herbal blends (77, 93).

Specific compounds also reflected ratio-dependent therapeutic potential. Phenylpropanoids (e.g., eugenol) and sesquiterpenes (e.g., α-bisabolol, nerolidol) in the 1:1 ratio align with known antimicrobial and antioxidant properties of tea and *Mentha* essential oils (94, 95). The detection of linoleic acid and β-caryophyllene in the 1:2 ratio suggests enhanced anti-inflammatory activity, consistent with reports that polyunsaturated fatty acids and sesquiterpenes modulate inflammatory pathways (96, 97). In contrast, the dominance of eucalyptol and camphor in the 2:1 ratio supports their traditional use in respiratory therapies due to expectorant and bronchodilatory effects (98). However, the detection of industrial contaminants such as DEHP and siloxanes raises concerns about possible extraction or storage contamination, underscoring the need for strict quality control (99). Optimizing both extraction conditions and blend ratios is therefore essential to maximize therapeutic benefits while minimizing toxic risks. Future studies should further investigate synergistic mechanisms among these compounds and validate their bioactivity through *in vitro* and *in vivo* assays.

#### 4.1.4 Bioactive richness among tea–*Mentha* blends

Comparative analysis highlighted the critical role of *Mentha* proportion in enhancing chemical diversity and functional potential. The 1 Tea + 2 *Mentha* ratio exhibited the highest richness (three compound classes), consistent with reports that *Mentha* species are abundant in monoterpenes and oxygenated compounds with antioxidant, antimicrobial, and anti-inflammatory activities (90, 92). Key constituents such as α-pinene and caryophyllene are well documented for antimicrobial and anti-inflammatory effects, while oxygenated compounds, including eugenol and bisabolol, provide strong antioxidant activity (83).

The synergistic presence of these compound classes in *Mentha*-rich blends likely enhances functional efficacy through additive or synergistic interactions, as demonstrated in studies on green tea–peppermint combinations (82). In contrast, blends with lower *Mentha* content showed reduced richness and fewer therapeutic effects, underscoring the importance of optimizing blend composition for nutraceutical and functional beverage development. These findings support the formulation of *Mentha*-enriched teas as promising candidates for health-promoting applications.

#### 4.1.5 Best combination of Chinese tea–*Mentha* blends

Increasing the proportion of *Mentha* in tea blends markedly enhanced bioactive richness and therapeutic potential. The 1 Tea + 2 *Mentha* ratio achieved the highest weighted richness index and compound class diversity, reflecting the phytochemical complexity of *Mentha* species. Rich in phenolics, flavonoids, and terpenoids, *Mentha* contributes potent antioxidant, antimicrobial, and anti-inflammatory effects (92). Its essential oils, particularly those containing α-pinene and caryophyllene, exhibit broad antimicrobial activity and anti-inflammatory properties (79, 90). Oxygenated compounds such as eugenol and bisabolol further enhance antioxidant capacity, reducing oxidative stress and supporting health-promoting effects (83).

These synergistic interactions explain why *Mentha*-rich blends display greater functional diversity than tea-dominant ratios. Previous studies also highlight the role of *Mentha* teas in gastrointestinal health, immune modulation, and cardiovascular protection (90, 92). Collectively, these findings suggest that optimizing blend ratios to maximize *Mentha*-derived bioactives offers strong potential for nutraceuticals, functional beverages, and natural antimicrobial formulations.

### 4.2 *In silico* interactions and networks of compounds and their target gene expression

Because GC-MS selectively detects volatile and semi-volatile metabolites, our analytical results are limited to compounds within this chemical class. Non-volatile tea constituents such as catechins, theaflavins, thearubigins, and L-theanine were not detected in this study and are discussed only as background information. All transcriptomic mappings and compound–gene associations were therefore restricted exclusively to GC-MS–identified volatiles. As a result, the integrative nutrigenomic analysis reflects a predictive and hypothesis-generating exploration of volatile phytochemicals, rather than a comprehensive assessment of all bioactive components typically found in tea.

#### 4.2.1 Network of compound–tissue and gene expression in human circulatory and respiratory tissues

Our analysis revealed strong compound–gene associations, particularly with antioxidant and stress-response genes such as *CAT*, *GPX1*, *HMOX1*, and *NQO1*. These results are consistent with previous studies showing that polyphenols and fatty acids restrain the predictive transcriptional networks regulating oxidative stress and inflammation. For instance, Kang and Kim (100) reported that bioactive compounds regulate cellular redox balance and histone acetylation, activating transcription factors such as NRF2 and SIRT1 to mitigate oxidative stress and inflammatory signaling. Similarly, Lee et al. (101) emphasized the role of naturally derived compounds in reducing ROS and inflammatory mediators in chronic conditions, reinforcing the therapeutic potential of these molecules. Our observation that linoleic and alpha-linolenic acids strongly influence antioxidant genes aligns with studies on lipid-derived bioactives, which have been shown to alter oxidative stress and lipid metabolism pathways (102). Moreover, the connectivity of terpenoids such as beta-myrcene and alpha-pinene to detoxification genes parallels findings from Luo et al. (103), who demonstrated that terpenoid biosynthesis in *Amomum tsaoko* involves transcription factors like MYB and WRKY, contributing to antioxidant and anti-inflammatory activity.

Polyphenols, including EGCG, quercetin, and resveratrol, are well documented for modulating gene expression through NF-κB and Nrf2 pathways, as well as exerting epigenetic effects such as microRNA regulation (104, 105). Their strong associations with antioxidant genes in our study corroborate these mechanisms. Collectively, these findings underscore the pleiotropic role of bioactive compounds in stress adaptation and metabolic homeostasis, supporting the need for multi-omics approaches to validate synergistic effects (106, 107). Within the interaction network, EGCG showed the highest connectivity, linking to *CAT*, *NQO1*, and *SOD1*, consistent with its potent antioxidant and anti-inflammatory effects via NF-κB and Nrf2 signaling (108, 109). EGCG reduces ROS and cytokines, protecting against oxidative damage and improving metabolic balance (110). Resveratrol and quercetin also showed strong associations with lipid metabolism and inflammatory genes, consistent with their regulation of AMPK and NF-κB pathways (111, 112). Quercetin, in particular, was linked to *GPX1* and *HMOX1*, reflecting its dual antioxidant and anti-inflammatory roles (113). Its regulatory effects on inflammatory mediators and lipid signaling pathways suggest a dual role in mitigating oxidative stress and metabolic dysfunction (114). Other compounds, such as vitamin E and curcumin, demonstrated moderate connectivity, reinforcing their established roles in redox regulation through Nrf2 and PPAR-γ activation (111, 115).

These results align with recent transcriptomic and metabolomic studies. For instance, Luo et al. (103) showed that curcuminoids and terpenoids regulate antioxidant biosynthesis genes, while Xu et al. (107) reported triterpenoid-mediated activation of transcription factors and cytochrome P450 enzymes in *Ganoderma lucidum*. Similar tissue-specific regulation of flavonoid-related genes in *Citrus grandis* and *Paeonia lactiflora* (106, 107) mirrors our findings on quercetin’s influence on detoxification and lipid metabolism.

The interplay between oxidative stress, lipid metabolism, and inflammation is central to chronic disease pathogenesis (116, 117). Compounds with strong connectivity to these pathways may therefore represent promising therapeutic candidates. Tissue-specific expression patterns further support this: antioxidant genes (*HMOX1*, *CAT*, *GPX1*, *NQO1*) were highly expressed in oxidative stress-sensitive tissues such as liver, kidney, and heart, consistent with prior reports on polyphenol-mediated activation of detoxification pathways (104, 118). Lipid-derived compounds such as linoleic and oleic acids were linked to metabolic genes (*PPARG*, *ACACA*) in adipose and pancreatic tissues (119, 120), while terpenoids were associated with detoxification genes in muscle tissue (103).

Comparative transcriptomic studies in medicinal plants reinforce these insights. Xu et al. (107) reported tissue-specific accumulation of flavonoids and monoterpene glycosides in *Paeonia lactiflora*, while Wang et al. (121) demonstrated MYB-mediated regulation of flavonoid biosynthesis in *Panax quinquefolius*. Together, these findings confirm that bioactive compounds exert pleiotropic effects on transcriptional networks governing oxidative stress, lipid metabolism, and inflammation. Future research should integrate multi-omics approaches to validate these associations and explore synergistic interactions among compound classes (122, 123).

#### 4.2.2 Network of compound–tissue and gene expression in various humans based on the Illumina Body Map 2—FBKM

The tissue-specific expression patterns observed in this study provide important insights into the functional roles of target genes across human tissues. Antioxidant-related genes such as *CAT*, *GPX1*, and *HMOX1* exhibited high expression in metabolically active tissues (liver, kidney, heart), consistent with their established roles in oxidative stress regulation. These findings align with previous transcriptomic analyses demonstrating that antioxidant enzymes are highly expressed in organs with elevated metabolic activity to counteract reactive oxygen species (ROS) (115, 118). Similarly, detoxification genes such as *NQO1* showed moderate expression in muscle and brain, supporting earlier reports that these tissues rely on phase II detoxification pathways for neuroprotection and metabolic adaptation (122).

Genes involved in lipid metabolism, including *PPARG* and *ACACA*, displayed tissue-specific expression in adipose and pancreatic tissues, which agrees with prior studies highlighting their roles in adipogenesis and glucose homeostasis (119, 120). The observed expression of *INS* in pancreatic tissue further corroborates its well-documented role in endocrine regulation. These patterns mirror findings from large-scale transcriptomic projects, such as *GTEx*, which emphasize the importance of tissue context in interpreting gene function (123).

Comparative studies on bioactive compounds have shown that polyphenols (e.g., EGCG, quercetin, and resveratrol) and fatty acids restrain these same pathways through transcriptional regulation. For example, *EGCG* and quercetin activate *Nrf2* signaling, enhancing antioxidant gene expression, while fatty acids influence *PPARG*-mediated lipid metabolism (100, 104). Our results complement these findings by providing baseline tissue-specific expression profiles, which can guide future investigations into compound-driven modulation of these genes.

The present study highlights the strong connectivity between bioactive compounds and antioxidant-related genes, particularly *CAT*, *HMOX1*, *GPX1*, *NQO1*, and *SOD1*, which exhibited 8–10 links each. These genes are central to the cellular defense against oxidative stress, functioning as primary enzymatic antioxidants. Our findings align with previous reports emphasizing the indispensable role of *SOD*, *CAT*, and *GPX* as first-line defense enzymes in neutralizing reactive oxygen species (ROS) and maintaining redox homeostasis (124). The high connectivity of *CAT* and *HMOX1* observed in our network suggests that these genes may serve as critical hubs for mediating the protective effects of polyphenols and fatty acids, consistent with earlier studies demonstrating their upregulation under oxidative stress conditions (125).

Polyphenolic compounds such as quercetin and resveratrol, which showed broad interactions in our network, have been previously reported to activate antioxidant response pathways through NRF2-mediated signaling, leading to increased expression of *SOD*, *CAT*, and *HMOX1* (124). Similarly, fatty acids like linoleic and alpha-linolenic acids have been implicated in modulating oxidative stress by influencing lipid peroxidation and enhancing antioxidant enzyme activity (126). Our observation that all compounds exhibited equal connectivity (five links each) suggests a synergistic effect, supporting the hypothesis that dietary antioxidants collectively contribute to redox balance rather than acting in isolation. Furthermore, genetic polymorphisms in antioxidant genes such as *MnSOD*, *CAT*, and *GPX1* have been associated with altered susceptibility to oxidative stress-related disorders, including cardiovascular disease and obesity (127, 128). These findings underscore the importance of considering genetic variability when interpreting compound–gene interactions. While our study focused on connectivity patterns, future research should integrate polymorphism data and expression profiles to better understand individual responses to antioxidant interventions.

The database-derived sex-specific expression patterns of antioxidant genes and their association with bioactive compounds suggest distinct regulatory mechanisms in male and female tissues. In male individuals, the liver and adrenal gland exhibited high expression of *SOD2*, *CAT*, and *GPX1*, which correlated with compounds such as resveratrol and curcumin. This aligns with previous findings that polyphenols like resveratrol enhance hepatic antioxidant capacity by activating *AMPK* and reducing oxidative stress, particularly in male models where metabolic stress induces higher *AMPK* activity (112). Conversely, female tissues such as adipose and kidney showed elevated expression of *HMOX1* and *SOD2*, associated with vitamin E and linoleic acid, supporting evidence that estrogen-mediated signaling upregulates mitochondrial antioxidant enzymes, conferring greater oxidative protection in females (129). These sex-specific differences may explain why females generally exhibit stronger resilience to oxidative damage and longer lifespan compared to males.

The compound associations observed in this study also reflect tissue-specific metabolic roles. For instance, vitamin E and linoleic acid linked to adipose tissue are consistent with reports that these compounds restrain lipid peroxidation and improve adipose function under oxidative stress (130). Similarly, resveratrol and quercetin associated with liver tissues corroborate previous studies showing their ability to activate detoxification pathways and reduce hepatic lipid accumulation through PPAR signaling (131). These findings emphasize the therapeutic potential of polyphenols and fatty acids in targeting oxidative stress-related disorders, with implications for personalized nutrition strategies that consider sex-specific metabolic profiles.

#### 4.2.3 Network of compound–tissue and gene expression in the human nervous system

The predictive differential expression of antioxidant and metabolic genes across nervous system tissues highlights their critical roles in maintaining neuronal homeostasis. Antioxidant enzymes such as *SOD1*, *GPX1*, and *CAT* form the first line of defense against reactive oxygen species (ROS), mitigating oxidative stress that is particularly detrimental in the brain due to its high metabolic activity and lipid content (124, 132). Dysregulation of these enzymes has been implicated in neurodegenerative disorders, including Alzheimer’s disease and amyotrophic lateral sclerosis, where oxidative imbalance accelerates neuronal damage (133, 134). Similarly, lipid metabolism genes such as *FDFT1* and *ACAT1* exhibited strong expression in white matter regions, suggesting their involvement in cholesterol biosynthesis and membrane integrity, processes essential for synaptic function and neural repair (135, 136). The elevated expression of NPY in limbic structures aligns with its established role in energy homeostasis, stress adaptation, and neuroimmune modulation (137, 138). Furthermore, *NFKBIA* and *RELA*, key components of the NF-κB pathway, were expressed in regions associated with autonomic regulation, supporting their involvement in neuroinflammatory signaling and neuronal survival under stress conditions (139, 140). Cooperatively, these findings underscore the interplay between oxidative stress defense, lipid metabolism, and signaling pathways in sustaining neural function and resilience. Future research should explore therapeutic strategies targeting these gene networks to mitigate neurodegenerative processes and enhance neuroprotection.

The associative compound–gene interactions highlight the potential of plant-derived bioactive molecules in modulating neural antioxidant and metabolic pathways. Essential oil constituents such as camphene, geraniol, and vitamin E demonstrated strong associations with genes encoding antioxidant enzymes, including *SOD1*, *GPX1*, and *CAT*, suggesting their role in mitigating oxidative stress—a key contributor to neurodegenerative processes (141, 142). These compounds likely enhance endogenous antioxidant defenses by upregulating gene expression and activating enzymatic pathways, thereby reducing reactive oxygen species and neuronal damage (143). Similarly, terpenoids such as alpha-pinene and caryophyllene exhibited robust links to lipid metabolism genes like *FDFT1* and *ACAT1*, indicating their involvement in maintaining membrane integrity and cholesterol homeostasis, which are critical for synaptic function and neuroprotection (144, 145). The neuroprotective effects of these phytochemicals may also extend to anti-inflammatory and anti-apoptotic mechanisms, as evidenced by their capacity to modify NF-κB signaling components such as *RELA* and *NFKBIA*, thereby attenuating neuroinflammation and promoting neuronal survival (142, 146). Collectively, these findings underscore the therapeutic promise of phytochemicals as modulators of gene networks implicated in oxidative stress and lipid metabolism, offering a natural strategy for enhancing neural resilience and preventing neurodegenerative disorders.

The predictive observed heterogeneity in gene expression across nervous system tissues underscores the complexity of neuroprotective and metabolic pathways. Antioxidant enzymes such as *SOD1*, *GPX1*, and *CAT* play pivotal roles in mitigating oxidative stress by neutralizing reactive oxygen species (ROS), a process essential for neuronal survival given the brain’s high metabolic demand and lipid-rich environment (132, 147). Dysregulation of these enzymes has been linked to neurodegenerative disorders, including amyotrophic lateral sclerosis and Alzheimer’s disease, highlighting their therapeutic relevance (134, 148). Similarly, metabolic regulators such as *FDFT1* and *ACAT1*, which are involved in cholesterol and lipid biosynthesis, exhibited strong expression in white matter regions, suggesting a role in maintaining membrane integrity and synaptic function (131, 149). The elevated expression of NPY in limbic structures aligns with its established function in energy homeostasis and stress adaptation, while *NFKBIA* and *RELA* expression patterns reflect the involvement of NF-κB signaling in neuroinflammation and autonomic regulation (138, 140). Collectively, these findings support the hypothesis that antioxidant defense and lipid metabolism genes are central to neuronal resilience, whereas signaling molecules such as NF-κB components and neuropeptides mediate adaptive responses to physiological and pathological stimuli. Future studies should explore how these gene networks interact under stress conditions and whether targeted modulation can enhance neuroprotection.

#### 4.2.4 Network of compound–tissue and gene expression in human skeletal immune digestive tissues

The observed associative differential gene expression in response to various compounds underscores the complexity of plant metabolic regulation and signaling pathways. Our findings indicate that linolenic acids and α-linolenic acid exert strong regulatory effects on genes such as *ESR1*, *CTSD*, and *LEP*, which aligns with previous studies demonstrating the role of α-linolenic acid as a precursor for jasmonic acid, a key phytohormone involved in stress signaling and transcriptional modulation (150, 151). The upregulation of these genes suggests that fatty acids not only serve structural and energy roles but also act as signaling molecules influencing transcriptional networks under stress conditions (152).

Similarly, phytol and related terpenoids showed significant effects on multiple genes, indicating their involvement in secondary metabolism and defense responses. Terpenoids are known to interact with transcription factors such as WRKY, MYB, and bHLH, which regulate terpene synthase genes and contribute to plant adaptation and aroma biosynthesis (153, 154). This supports the hypothesis that terpenoids function beyond ecological interactions, playing a role in modulating gene expression under biotic and abiotic stress.

The relatively lower impact of compounds like eucalyptol and beta-pinene suggests specificity in gene-compound interactions, possibly due to differences in compound structure and receptor affinity. Such specificity is consistent with previous transcriptomic analyses showing that only certain terpenoids significantly alter gene expression profiles (155). Collectively, these results highlight a coordinated regulatory network where fatty acids and terpenoids act as modulators of transcriptional activity, influencing pathways related to stress tolerance, lipid metabolism, and secondary metabolite biosynthesis. Understanding these interactions provides a foundation for metabolic engineering strategies aimed at enhancing stress resilience and nutritional quality in crops (156, 157).

The present investigation highlights the differential regulatory effects of plant-derived compounds on gene expression, revealing a strong association between polyunsaturated fatty acids and terpenoids with transcriptional modulation. Among the tested compounds, linolenic acid and α-linolenic acid exhibited the most pronounced influence on genes such as *ESR1*, *CTSD*, and *LEP*. This observation aligns with previous findings that unsaturated fatty acids serve as precursors for jasmonates, which are critical signaling molecules in stress response pathways (152). These fatty acids not only contribute to membrane fluidity and energy storage but also act as modulators of stress signaling and transcriptional regulation under abiotic and biotic stress conditions (152, 158). Similarly, terpenoids such as phytol and trans-phytol demonstrated significant effects on gene expression, supporting their role in secondary metabolism and defense. Terpenoids are known to interact with transcription factors like NAC, MYB, and bHLH, which regulate terpene synthase genes and enhance plant resilience against environmental stressors (159, 160). Recent multi-omics studies have confirmed that terpenoid biosynthesis is tightly linked to transcriptional networks and hormonal signaling, further emphasizing their importance in adaptive responses (154, 161).

The relatively lower impact of compounds such as geraniol, imidazole, and caffeine suggests specificity in compound-gene interactions, possibly due to structural differences and receptor affinity. This specificity is consistent with transcriptomic analyses showing that only certain terpenoids and fatty acids significantly alter gene expression profiles (162, 163). The hierarchical influence observed—where compounds with multiple links and higher expression values dominate transcriptional changes—underscores the potential of these metabolites as targets for metabolic engineering aimed at improving stress tolerance and crop quality (164, 165).

The database-inferred transcriptional patterns revealed strong tissue-specific expression patterns, underscoring the functional specialization of major physiological compartments. Liver tissues exhibited the most distinct signatures, with genes such as *ALB*, *APOC3*, and *FBP1* showing extreme enrichment. These findings are consistent with the liver’s central role in protein synthesis, lipid transport, and gluconeogenesis (166, 167). *APOC3*, in particular, is a key regulator of triglyceride metabolism and cardiovascular risk, highlighting the metabolic implications of hepatic gene expression (168).

Adipose depots demonstrated a coherent lipogenic and endocrine profile, marked by high expression of *LEP*, *ADIPOQ*, *LPL*, and *PPARG*. These genes govern adipokine secretion, lipid storage, and insulin sensitivity, reinforcing the concept of adipose tissue as an active endocrine organ rather than a passive fat reservoir (169, 170). Depot-specific expression patterns, such as elevated *ADIPOQ* in subcutaneous fat, align with previous evidence of developmental programming and functional heterogeneity among adipose depots (171). However, muscle compartments displayed strong expression of structural and contractile genes, notably *ACTA2* in smooth muscle and antioxidant enzymes (*SOD2*, *CAT*, *GPX1*) in skeletal muscle. These profiles reflect the dual roles of muscle in mechanical function and oxidative stress regulation (172, 173). The inflammatory hotspot of IL6 in smooth muscle suggests localized immune-metabolic crosstalk, consistent with its role in vascular remodeling and inflammation.

Immune-rich tissues exhibited context-dependent expression of regulators such as *TBX21*, *HMOX1*, *NFKBIA*, and *MYD88*, which orchestrate innate and adaptive immune responses. Elevated TBX21 in NK cells and lymphoid compartments aligns with its function as a master regulator of *Th1* differentiation and interferon signaling (174, 175). Similarly, *HMOX1* enrichment in the spleen reflects its cytoprotective role during oxidative stress and inflammation (163). In addition, vascular and lymphatic compartments were characterized by adhesion and guidance molecules, including *VCAM1*, *EPHB4*, *RHOA*, and *CDC42*, which mediate endothelial integrity, angiogenesis, and immune cell trafficking (176). These findings emphasize the interplay between vascular signaling and immune surveillance.

Finally, bone and endocrine tissues showed localized expression of *BGLAP* (osteocalcin) and neuropeptide *NPY*, linking skeletal remodeling with neuroendocrine regulation of energy metabolism (177, 178). Such cross-tissue signaling highlights the integration of metabolic and structural pathways. Collectively, these results reinforce the concept that tissue-specific gene expression is governed by specialized regulatory networks, enabling precise physiological functions. Understanding these patterns provides a foundation for targeted interventions in metabolic disorders, cardiovascular disease, and tissue engineering.

#### 4.2.5 Network of compound–tissue and gene expression in the human reproductive system

The strong expression response to phytol aligns with its role as a chlorophyll-derived diterpene and precursor for tocopherol (vitamin E) biosynthesis, which is essential for antioxidant defense and stress tolerance (179, 180). Phytol has also been implicated in signaling pathways such as ethylene-mediated stress responses, suggesting its involvement in modulating gene networks related to oxidative stress and lipid metabolism (180). While Oleamide, another highly connected compound, is known for its neuromodulatory properties and interaction with cannabinoid receptors, influencing sleep and stress regulation (181). Its linkage to *CDKN1A* and *ATM* indicates potential roles in cell cycle control and genomic stability. Similarly, linolenic acid, a precursor for jasmonic acid, showed strong associations with *TP53* and *HMGCR*, supporting its role in lipid metabolism and signaling pathways that regulate stress responses (152, 182).

Moderate expression levels observed for caffeine suggest its function as an adenosine receptor antagonist and epigenetic modulator rather than a direct transcriptional activator. Previous studies have demonstrated caffeine’s ability to influence DNA methylation and histone modifications, thereby altering gene expression patterns associated with metabolism and stress response (183, 184). However, its relatively lower impact compared to lipid-derived compounds indicates that its primary effects may be receptor-mediated or post-transcriptional.

These findings have important biological implications. Phytol and tocopherol pathways may serve as potential targets for enhancing antioxidant capacity and stress resilience, while oleamide signaling links lipid metabolism to neuroendocrine regulation, suggesting therapeutic potential for sleep and anxiety disorders. Similarly, linolenic acid metabolism is central to jasmonate signaling and defense pathways, relevant for stress physiology and metabolic health. In contrast, caffeine acts as a mild epigenetic modulator and neurostimulant, but its transcriptional influence appears less pronounced compared to lipid-derived compounds. Collectively, these results underscore the complexity of nutrigenomic interactions and highlight the need for integrative studies combining transcriptomics with functional assays to elucidate the effects of phytochemicals (152). These findings suggest that the predicted association with genes under compound influence is highly tissue-dependent, with lipid-derived molecules and terpenoids exerting stronger transcriptional effects compared to alkaloids like caffeine.

The Sankey diagram and heatmap analyses provide insights into the putative tissue expression patterns from public datasets of tea–*Mentha* phytochemicals on human reproductive genes. Among the top compounds, phytol exhibited the highest connectivity and expression levels, linking to *TP53*, *AKT1*, and *CASP3*. This observation aligns with phytol’s established role as a chlorophyll-derived diterpene and precursor for tocopherol (vitamin E), which is essential for antioxidant defense and stress tolerance (179, 180). Its strong association with *TP53* suggests involvement in DNA damage response and apoptosis pathways. The consistent presence of *TP53* across all compounds underscores its central role in stress signaling and apoptosis. This finding suggests that phytochemicals, particularly terpenoids and fatty acid derivatives, may exert pleiotropic effects on pathways governing cell survival, lipid metabolism, and oxidative stress. These results support the concept of nutrigenomics, where plant-derived compounds act as modulators of gene networks, influencing health and disease outcomes (181).

The heatmap analysis demonstrates substantial variability in gene expression across human tissues, highlighting tissue-specific transcriptional activity of key regulatory and metabolic genes. Genes such as *TP53*, *AKT1*, *CASP3*, and *CDKN1A* exhibited consistently high expression in multiple tissues, suggesting their central roles in stress response, apoptosis, and cell cycle regulation. These findings align with previous studies of Vousden and Prives (185), indicating *TP53* as a master regulator of genomic stability and apoptosis under stress conditions. Similarly, *AKT1* is widely recognized for its involvement in cell survival and metabolic signaling pathways (186), while *CASP3* plays a critical role in executing apoptosis (187). Conversely, genes such as *CYP1A2* and *VKORC1* showed low expression across most tissues, consistent with their specialized roles in xenobiotic metabolism and vitamin K cycle regulation, respectively (188, 189). The observed heterogeneity underscores the complexity of transcriptional regulation and suggests that tissue-specific gene expression patterns may influence physiological responses to environmental and dietary factors, including phytochemicals.

The clustering of highly expressed genes in certain tissues indicates functional specialization, particularly in pathways related to oxidative stress and metabolic adaptation. This supports the concept of nutrigenomics, where dietary compounds restrain gene expression to maintain cellular homeostasis (190). Because the present transcriptomic mapping is predictive and non-experimental, orthogonal validation is essential before drawing biological conclusions. The present results are regarded as hypothesis-generating signals that help prioritize compounds, tissues, and gene networks for future testing, not as evidence of confirmed nutrigenomic effects. Future studies integrating transcriptomics with metabolomics and epigenetic profiling could provide deeper insights into how phytochemicals influence gene networks across different tissue contexts. Finally, the strong network connectivity observed in this study corroborates previous evidence on the central role of enzymatic antioxidants in oxidative stress mitigation and highlights the potential of bioactive compounds as modulators of these pathways. These insights may inform strategies for personalized nutrition and therapeutic interventions targeting oxidative stress-related conditions.

## 5 Conclusion

This study demonstrates that the ratio of tea to *Mentha* significantly influences the chemical composition and functional potential of the resulting blend. Among the tested formulations, the 1:2 ratio emerged as the most promising, offering a superior balance of aromatic monoterpenes (e.g., eucalyptol and camphor) and health-promoting compounds such as palmitic and linoleic acids, phytol, and sesquiterpenes. These bioactives are associated with antioxidant, anti-inflammatory, and antimicrobial properties, supporting the development of functional beverages with enhanced nutraceutical value. Conversely, the 2:1 ratio, while rich in Eucalyptol, was dominated by methyl jasmonate, which may alter sensory characteristics and limit consumer appeal. The 1:1 ratio provided moderate levels of key compounds but lacked the bioactive density observed in 1:2. These findings underscore the importance of optimizing ingredient ratios to achieve both sensory quality and functional benefits, offering valuable insights for the food, beverage, and nutraceutical industries aiming to meet consumer demand for health-oriented products. Balanced blends, notably *1 Tea* + *1 Mentha 3*, offered a compromise with moderate diversity (65 compounds) and peak area (~95%), making them suitable for formulations targeting both functional and sensory attributes, while *2 Tea* + *1 Mentha 1* may be preferred for formulations prioritizing aroma and oxidative stability. Future studies should explore the bioactivity of these blends through antioxidant assays, antimicrobial tests, and sensory evaluations to validate their functional potential. The implications of these results extend to food preservation, pharmaceutical formulations, and natural antimicrobial agents. Future work should focus on quantitative bioassays to validate the predicted functional synergy and explore the mechanistic basis of interactions among compound classes.

## Data Availability

The original contributions presented in the study are included in the article/Supplementary material, further inquiries can be directed to the corresponding authors.

## Footnotes

1
https://ctdbase.org/

2
https://plotly.com/python/

3
https://matplotlib.org/stable/index.html

4
https://seaborn.pydata.org/

5
https://pandas.pydata.org/

6
https://numpy.org/

## Glossary

AAAS: Aladin WD repeat nucleoporin
AASDH: Aminoadipate-semialdehyde dehydrogenase
ABCA5: ATP binding cassette subfamily A member 5
ABCB1: ATP binding cassette subfamily B member 1
ABCB1A: ATP binding cassette subfamily B member 1A
ABCD3: ATP binding cassette subfamily D member 3
ABCE1: ATP binding cassette subfamily E member 1
ABHD12: Abhydrolase domain containing 12
ACACA: Acetyl-CoA carboxylase alpha
ACACB: Acetyl-CoA carboxylase beta
ACHE: Acetylcholinesterase
ACLY: ATP citrate lyase
ACOX1: Acyl-CoA oxidase 1
ACSL1: Acyl-CoA synthetase long chain family member 1
ACTA2: Actin alpha 2, smooth muscle
ADGRG1: Adhesion G protein-coupled receptor G1
AHR: Aryl hydrocarbon receptor
AKT1: AKT serine/threonine kinase 1
ALB: Albumin
APOC3: Apolipoprotein C3
AR: Androgen receptor
ATM: ATM serine/threonine kinase
ATP1A2: ATPase Na^+^/K^+^ transporting subunit alpha 2
ATP1A3B: ATPase Na^+^/K^+^ transporting subunit alpha 3B
ATP1B2B: ATPase Na^+^/K^+^ transporting subunit beta 2B
BAK1: BCL2 antagonist/killer 1
BAX: BCL2 associated X, apoptosis regulator
BCL2: BCL2 apoptosis regulator
BGLAP: Bone gamma-carboxyglutamate protein
BMP2: Bone morphogenetic protein 2
CACNA1AB: Calcium voltage-gated channel subunit alpha1 AB
CACNA1DA: Calcium voltage-gated channel subunit alpha1 DA
CASP3: Caspase 3
CASP9: Caspase 9
CAT: Catalase
CCL3: C-C motif chemokine ligand 3
CDC42: Cell division cycle 42
CDKN1A: Cyclin dependent kinase inhibitor 1A
CHRNA7: Cholinergic receptor nicotinic alpha 7 subunit
CHRNB4: Cholinergic receptor nicotinic beta 4 subunit
CNR2: Cannabinoid receptor 2
COL1A1: Collagen type I alpha 1 chain
CSF2: Colony stimulating factor 2
CTSD: Cathepsin
DCTSL: Cathepsin L
CXCL8: C-X-C motif chemokine ligand 8
CYP1A1: Cytochrome P450 family 1 subfamily A member 1
CYP2B1: Cytochrome P450 family 2 subfamily B member 1
DLG4: Discs large MAGUK scaffold protein 4
EPHB4: EPH receptor B4
ERBB4: Erb-b2 receptor tyrosine kinase 4
ESR1: Estrogen receptor 1
ESR2: Estrogen receptor 2
EST2: Esterase 2
FBP1: Fructose-bisphosphatase 1
FDFT1: Farnesyl-diphosphate farnesyltransferase 1
G6PC1: Glucose-6-phosphatase catalytic subunit 1
G6PD: Glucose-6-phosphate dehydrogenase
GABRA1: Gamma-aminobutyric acid type A receptor subunit alpha1
GNA15: G protein subunit alpha 15
GPX1: Glutathione peroxidase 1
GREB1: Growth regulation by estrogen in breast cancer 1
GRIN2D: Glutamate ionotropic receptor NMDA type subunit 2D
GSR: Glutathione-disulfide reductase
HMGCR: 3-hydroxy-3-methylglutaryl-CoA reductase
HMOX1: Heme oxygenase 1
IFNG: Interferon gamma
IL12RB2: Interleukin 12 receptor subunit beta 2
IL1B: Interleukin 1 beta
IL4: Interleukin 4
IL6: Interleukin 6
INS: Insulin
KLF6: Kruppel like factor 6
LEP: Leptin
LPL: Lipoprotein lipase
MAOA: Monoamine oxidase A
MYC: MYC proto-oncogene
MYD88: MYD88 innate immune signal transduction adaptor
NCOA2: Nuclear receptor coactivator 2
NFE2L2: Nuclear factor, erythroid 2 like 2
NFKB1: Nuclear factor kappa B subunit 1
NFKBIA: Nuclear factor of kappa light polypeptide gene enhancer in B-cells inhibitor alpha
NLRP3: NLR family pyrin domain containing 3
NOS2: Nitric oxide synthase 2
NPY: Neuropeptide Y
NQO1: NAD(P)H quinone dehydrogenase 1
NR1I2: Nuclear receptor subfamily 1 group I member 2
OGG1: 8-oxoguanine DNA glycosylase
OR5D18: Olfactory receptor family 5 subfamily D member 18
PGR: Progesterone receptor
PPARA: Peroxisome proliferator activated receptor alpha
PTGS2: Prostaglandin-endoperoxide synthase 2
PXN: Paxillin
RAP1A: RAS related protein 1A
RELA: RELA proto-oncogene, NF-kB subunit
RHOA: Ras homolog family member A
RHOB: Ras homolog family member B
SEC14L2: SEC14 like lipid binding 2
SLC27A4: Solute carrier family 27 member 4
SOD1: Superoxide dismutase 1
SOD2: Superoxide dismutase 2
SREBF2: Sterol regulatory element binding transcription factor 2
TBX21: T-box transcription factor 21
TLR4: Toll like receptor 4
TNF: Tumor necrosis factor
TP53: Tumor protein p53
UCP2: Uncoupling protein 2
UGT2B15: UDP glucuronosyltransferase family 2 member B15
VCAM1: Vascular cell adhesion molecule 1
VCL: Vinculin
VIP: Vasoactive intestinal peptide
VKORC1: Vitamin K epoxide reductase complex subunit 1
VKORC1L1: Vitamin K epoxide reductase complex subunit 1 like 1
