# Supplementary figures and images for: Integrative phytochemical profiling and in silico nutrigenomic predictions of Chinese tea–Saudi Mentha longifolia blend formulations
Source: Front Nutr. 2026 Mar 27;13:1753616. doi: 10.3389/fnut.2026.1753616 (PMC13065726; doi:10.3389/fnut.2026.1753616)

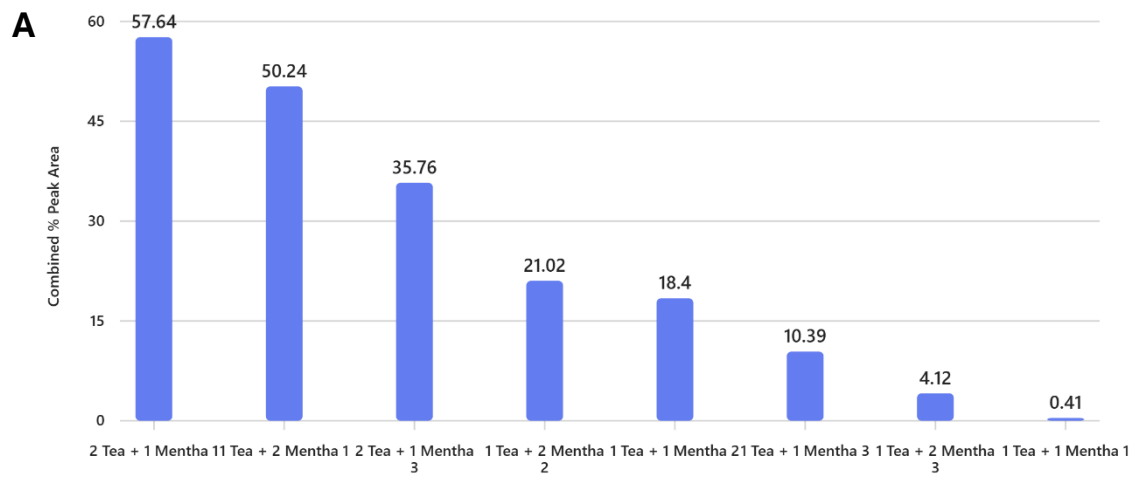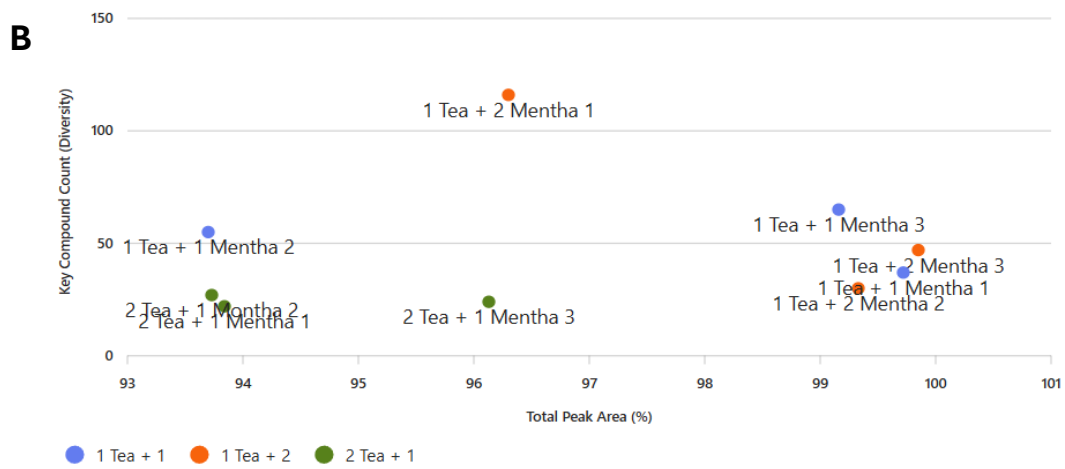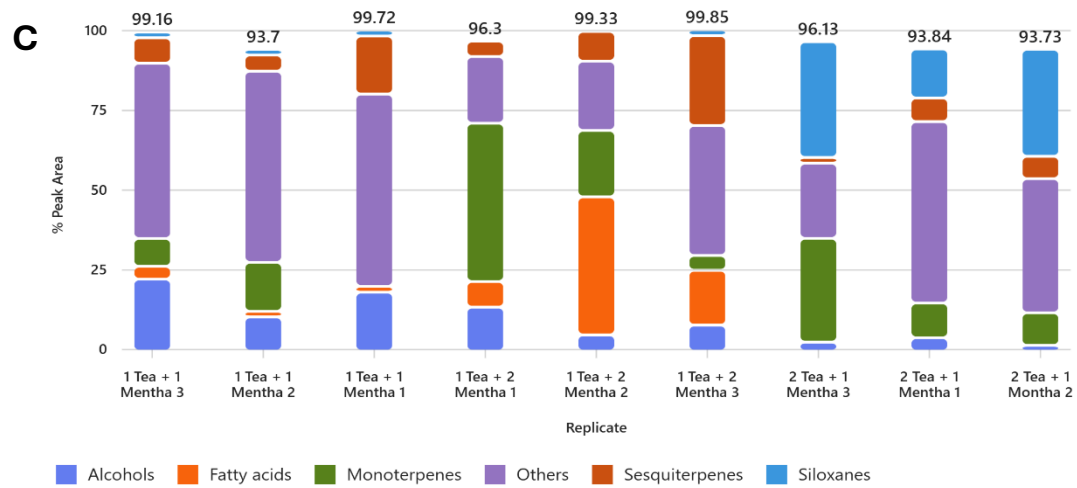

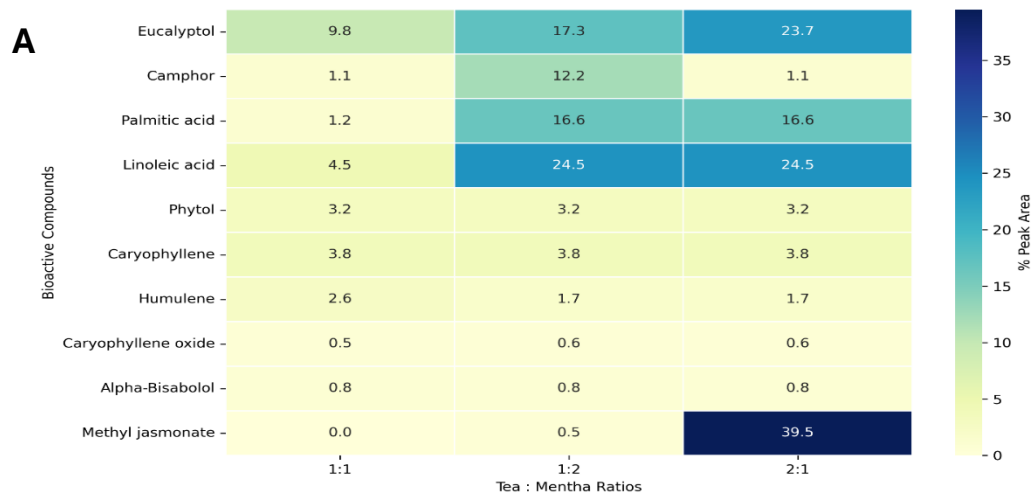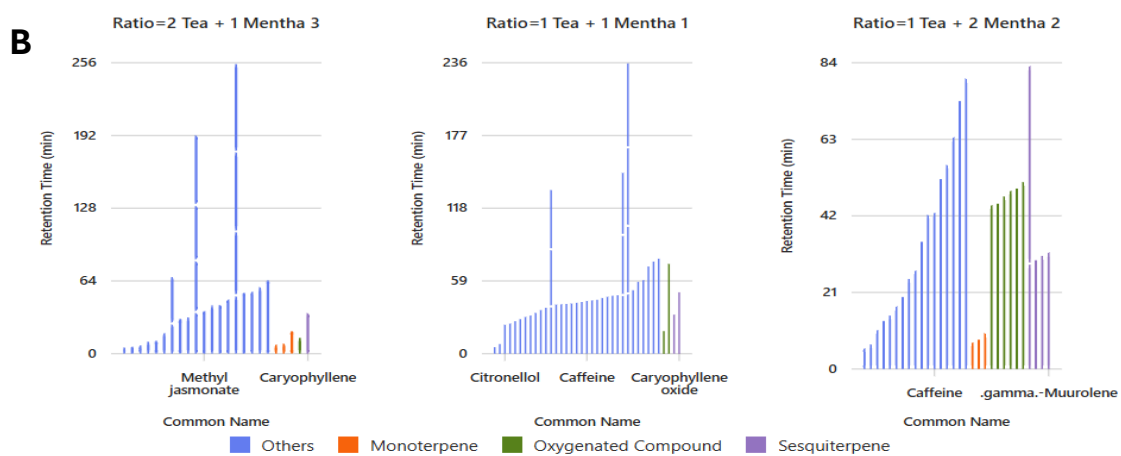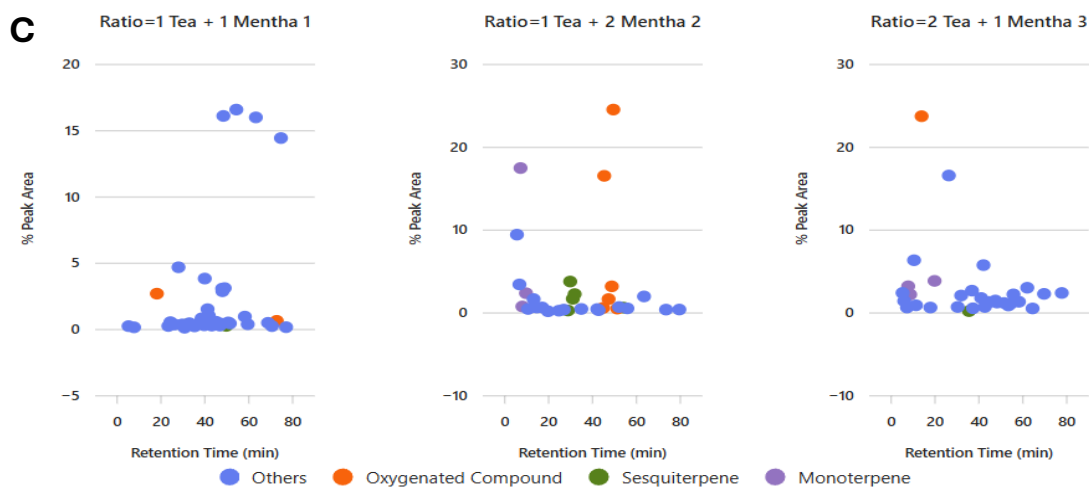

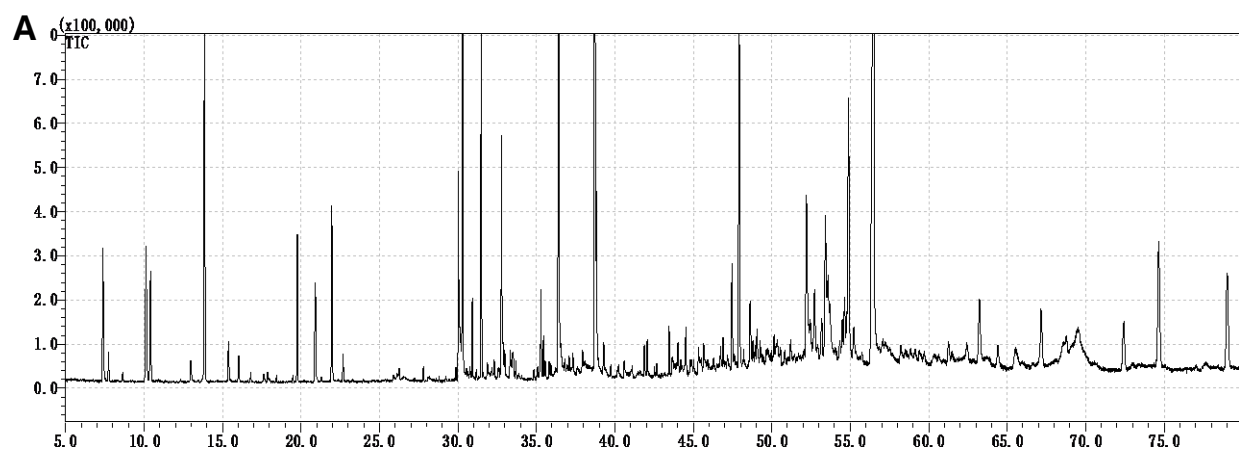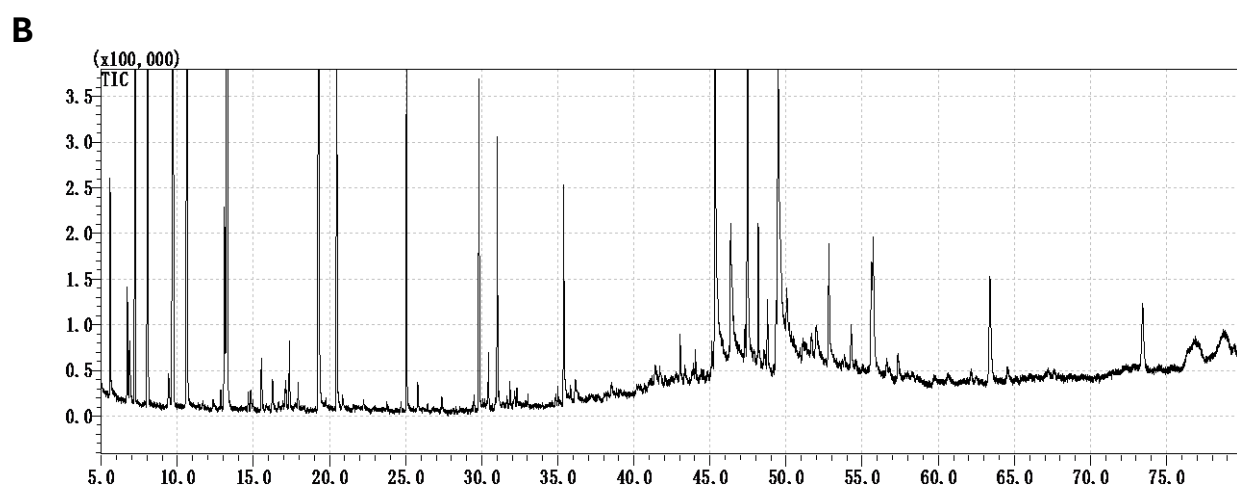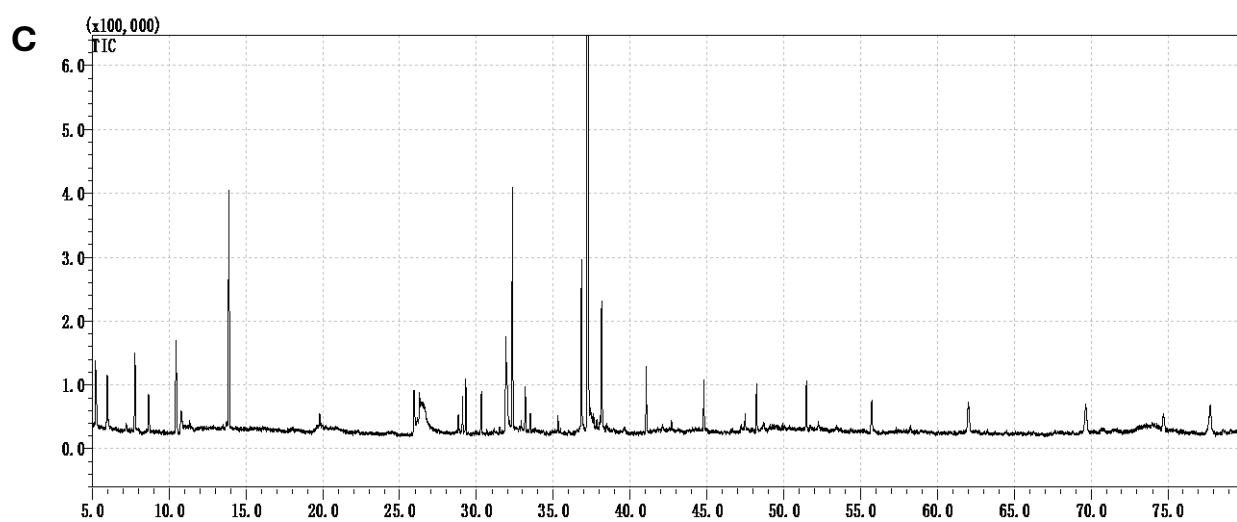

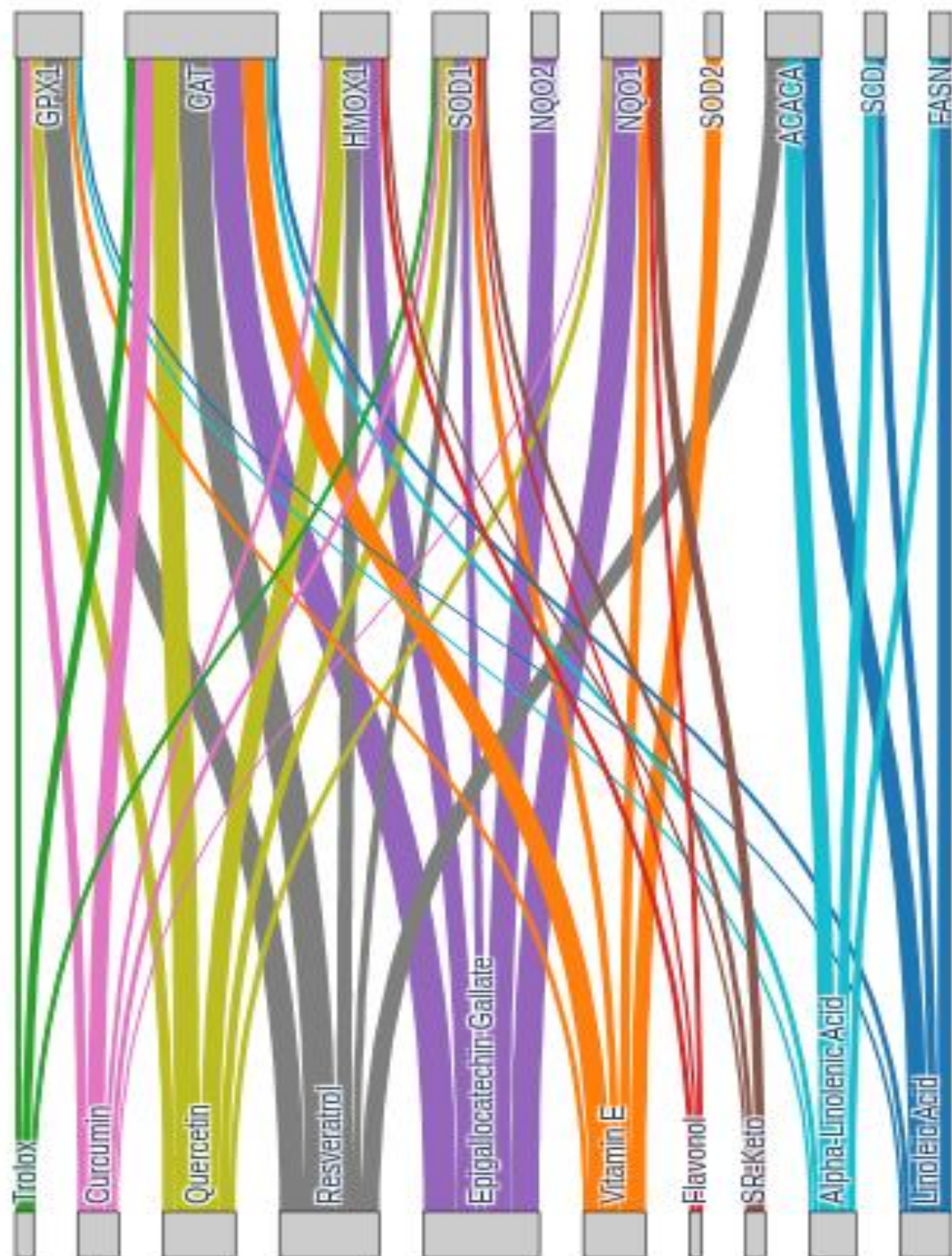

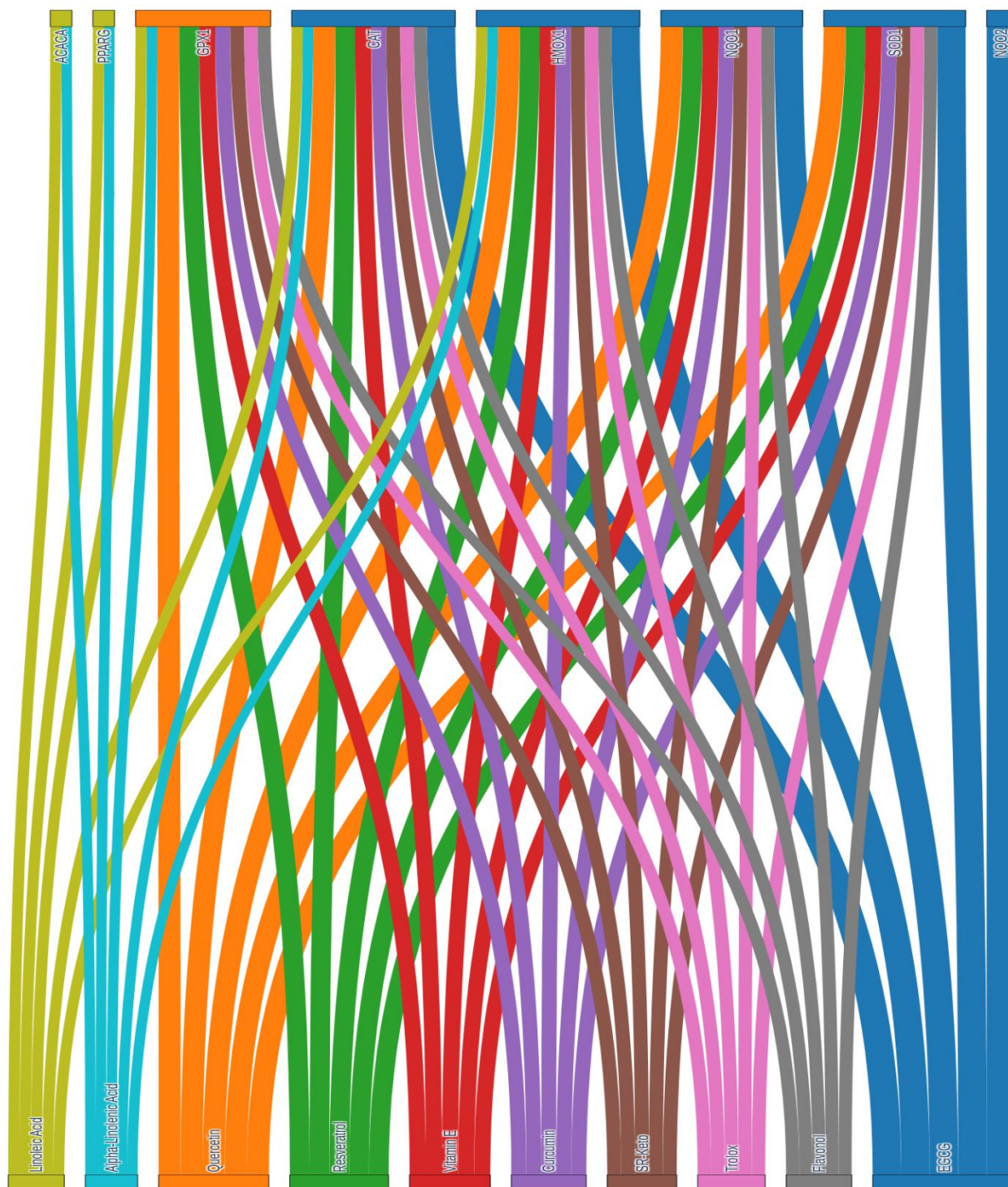

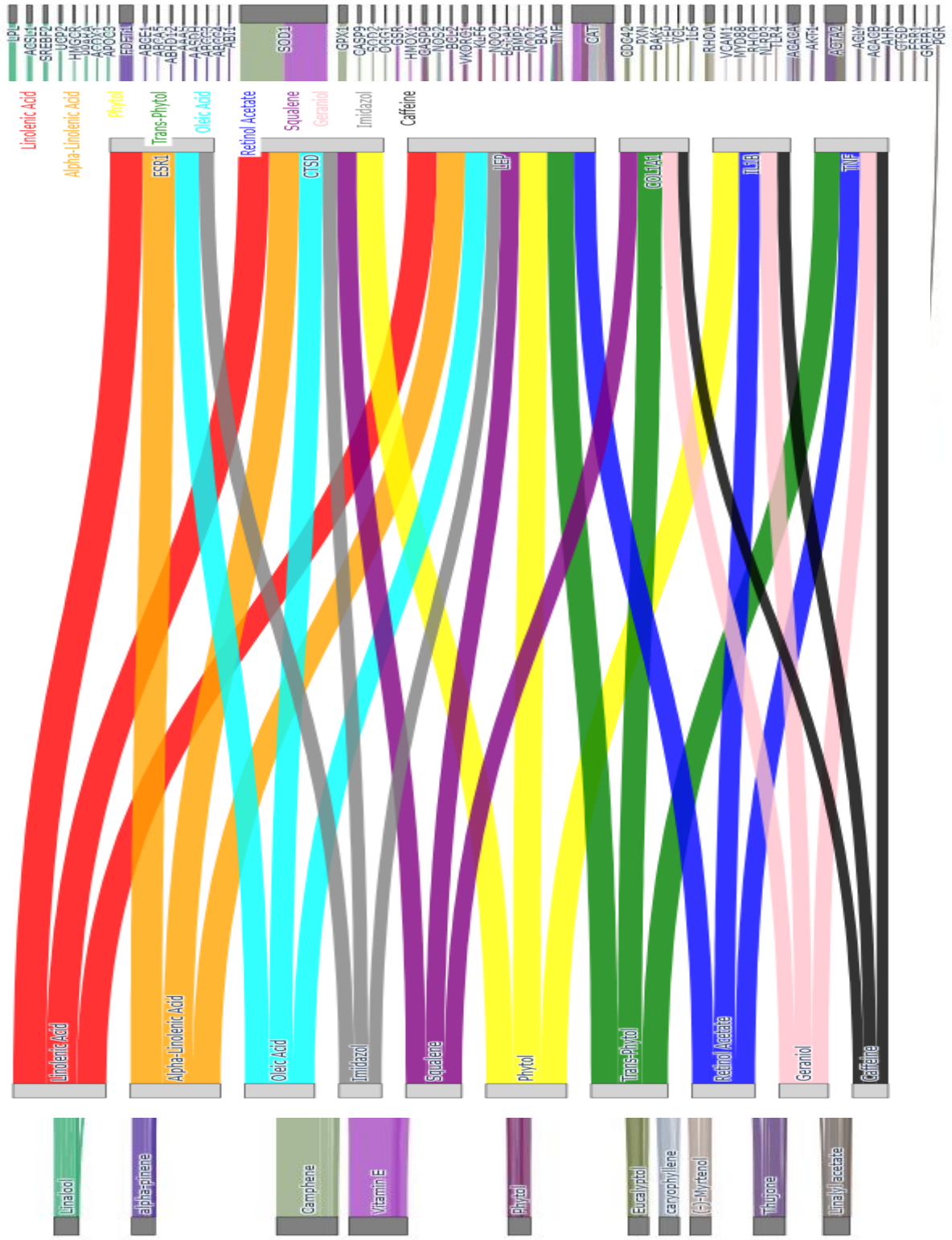

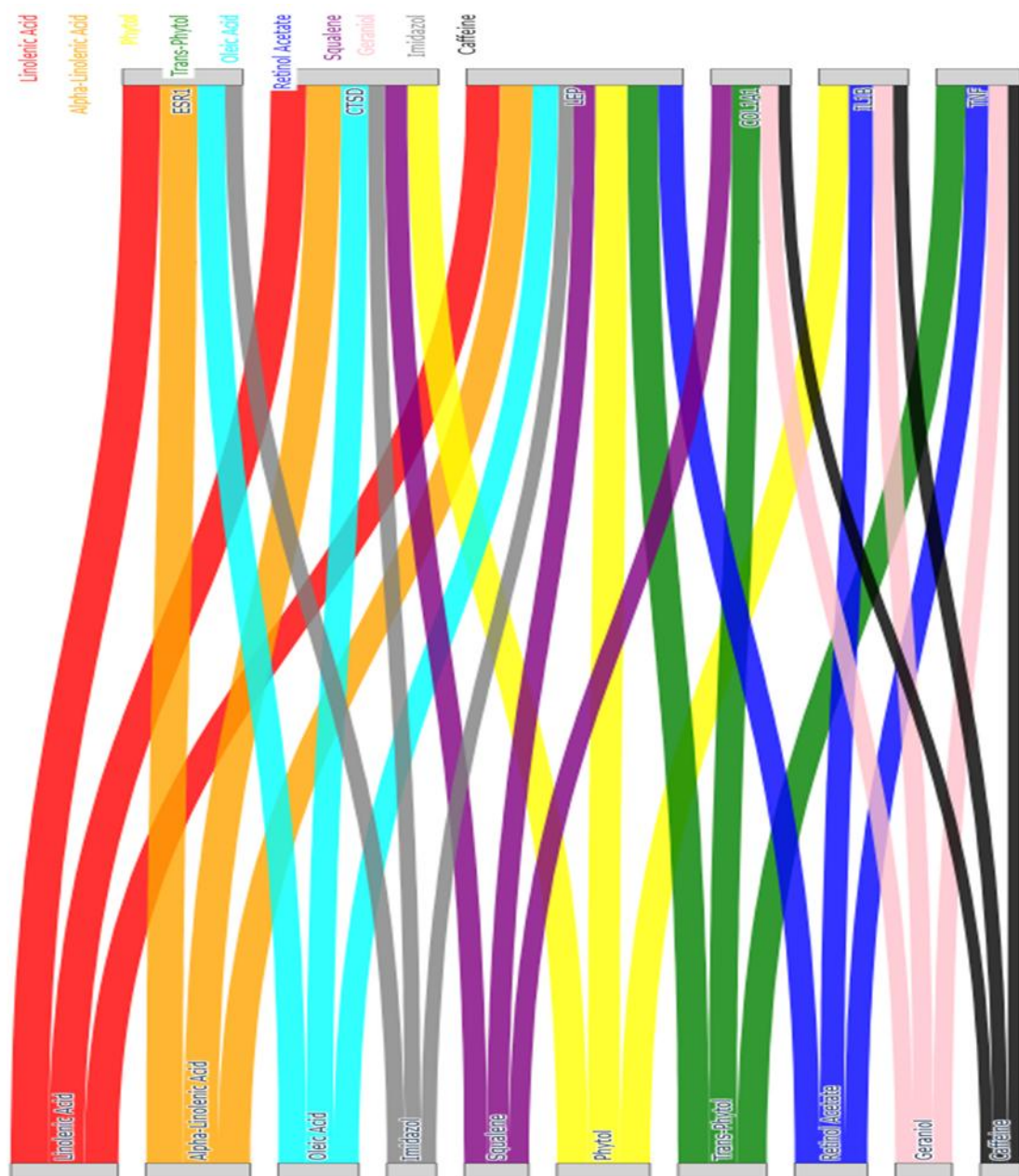

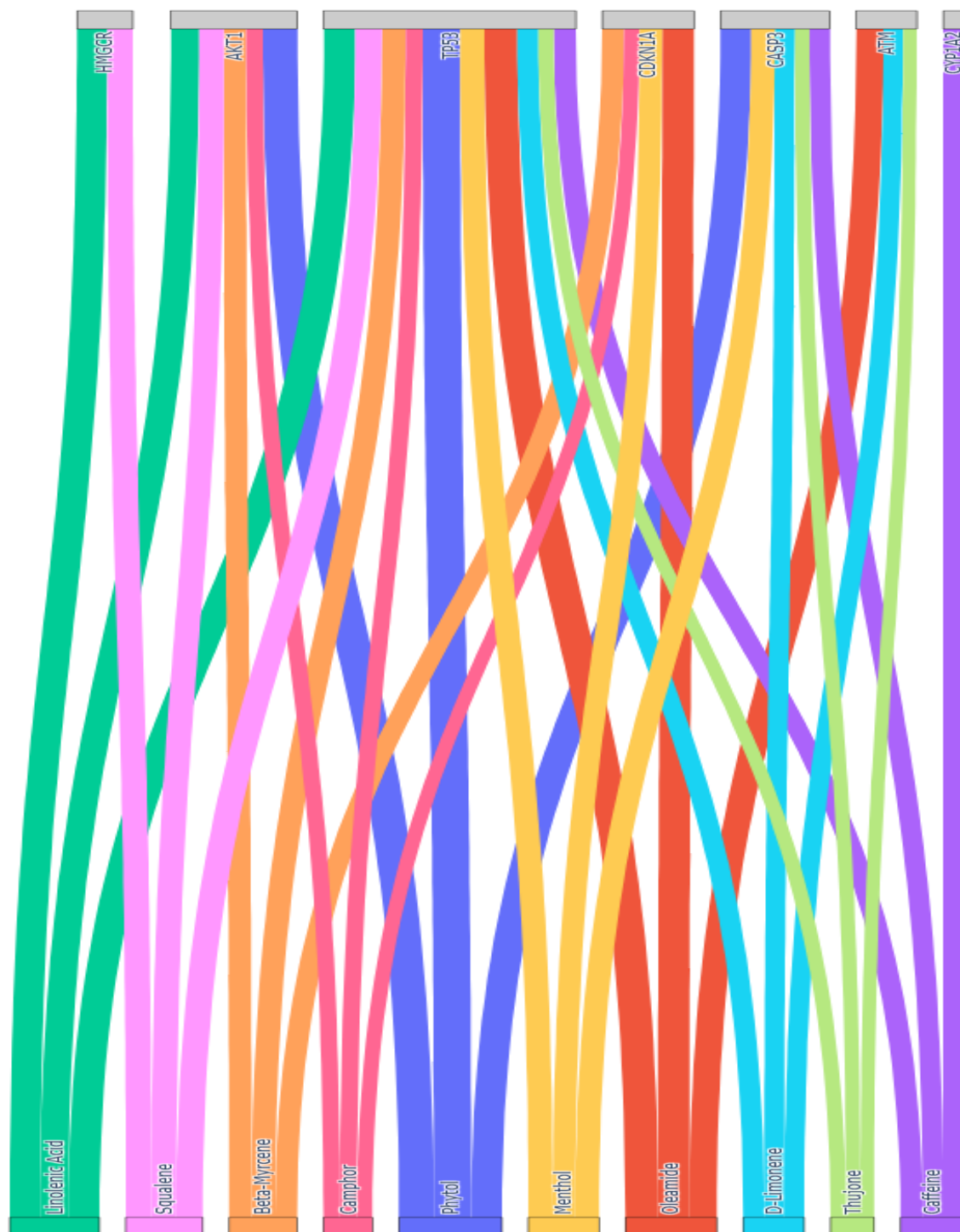

Supplement: Supplementary file 1 [file Supplementary_file_1.zip › Clean Revised Supplementary Figures 1-8.pdf]

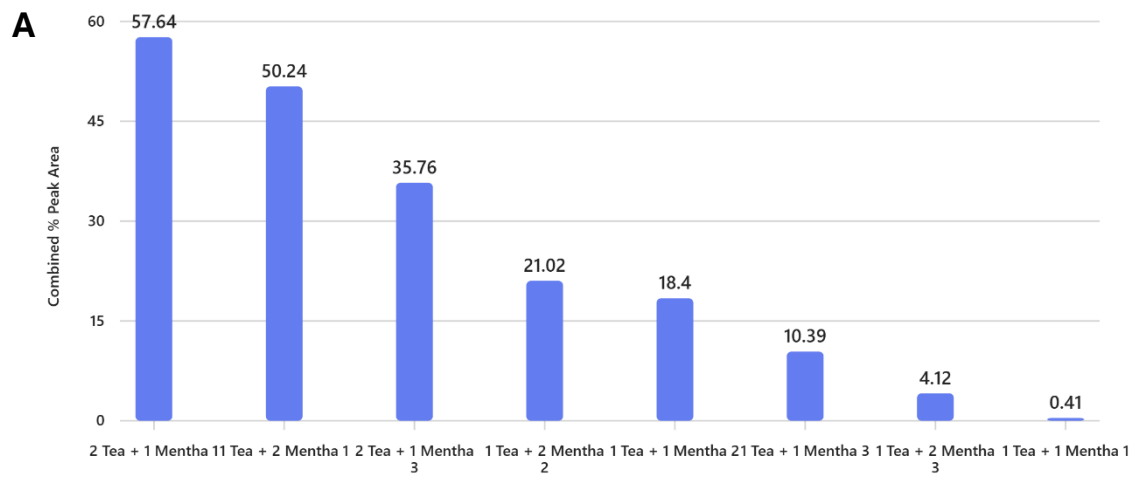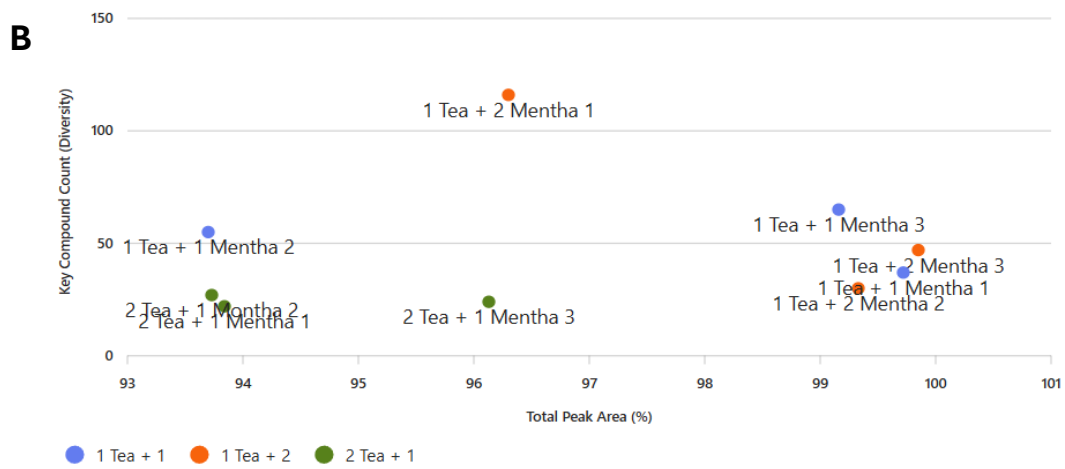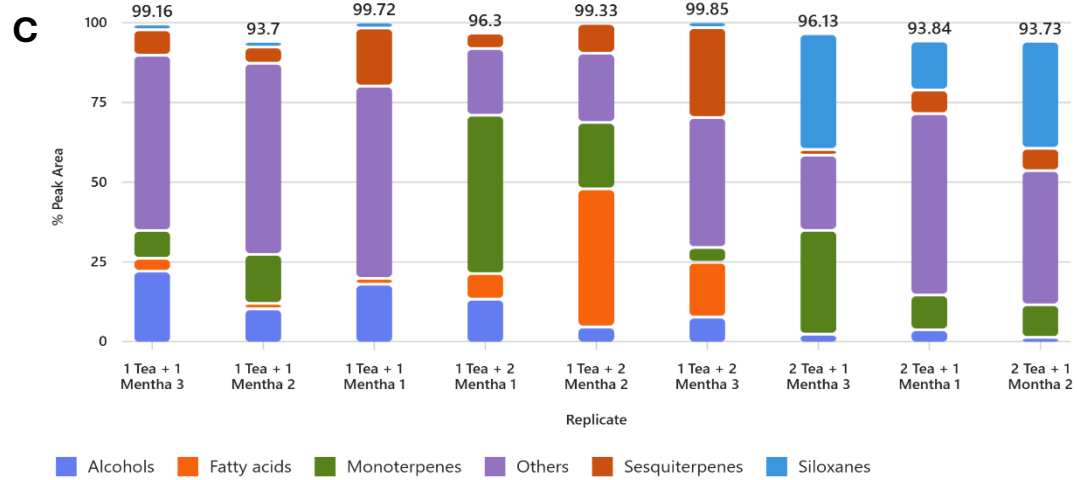

Supplement: Supplementary file 1 [file Supplementary_file_1.zip › Clean Revised Supplementary Figures_1.pdf]

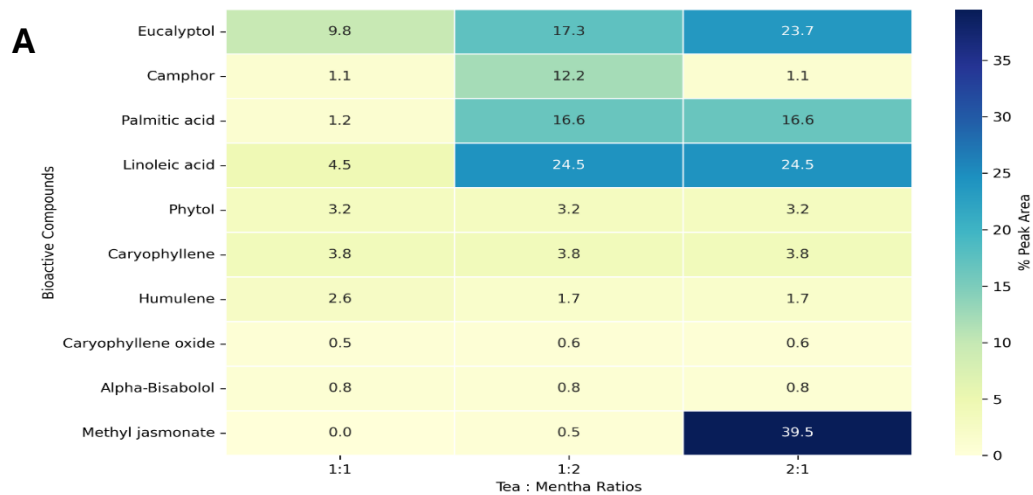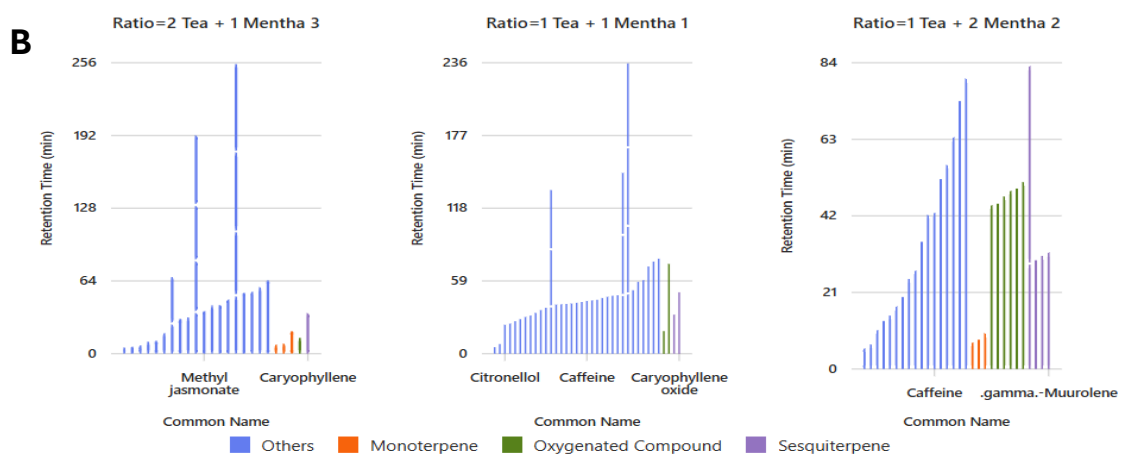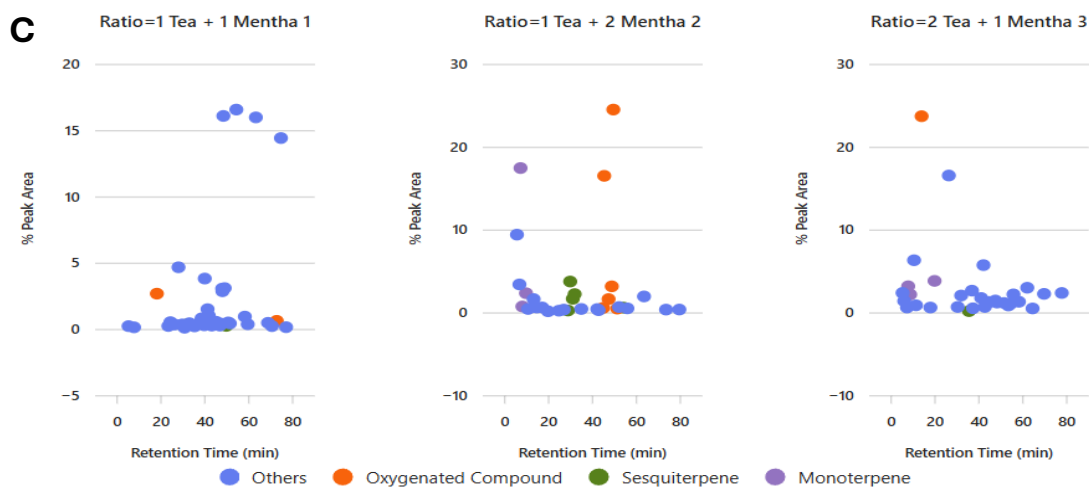

Supplement: Supplementary file 1 [file Supplementary_file_1.zip › Clean Revised Supplementary Figures_2.pdf]

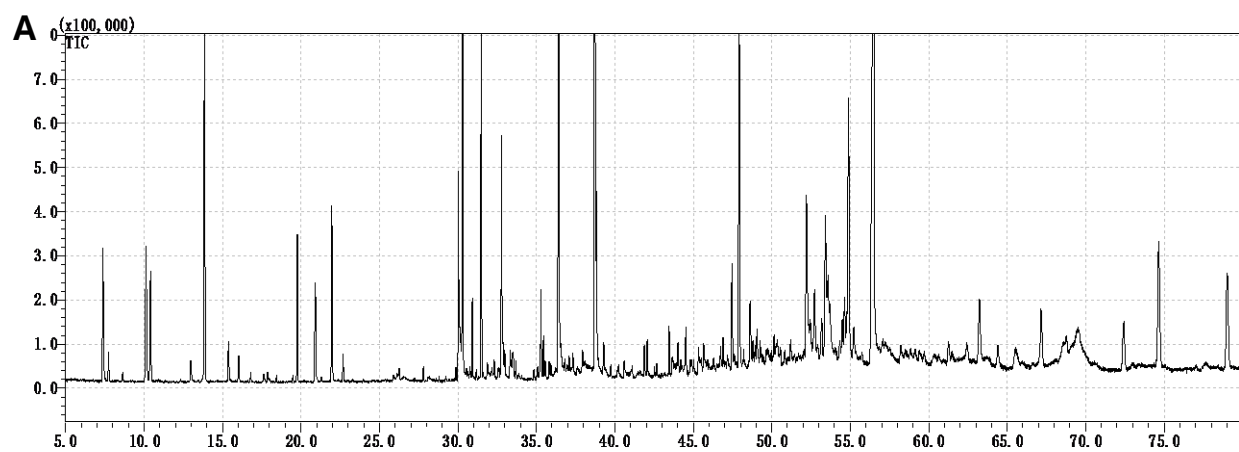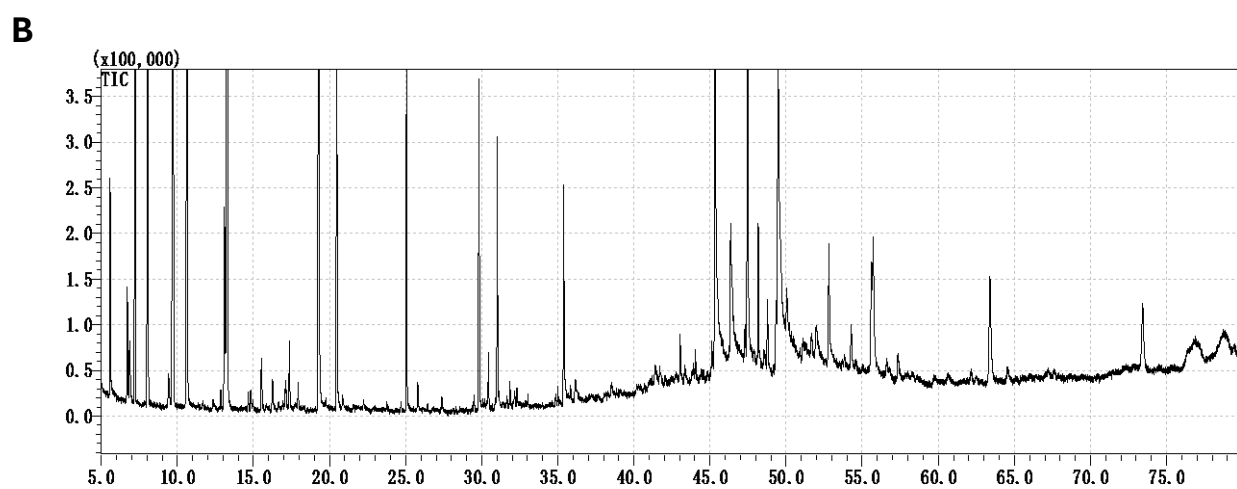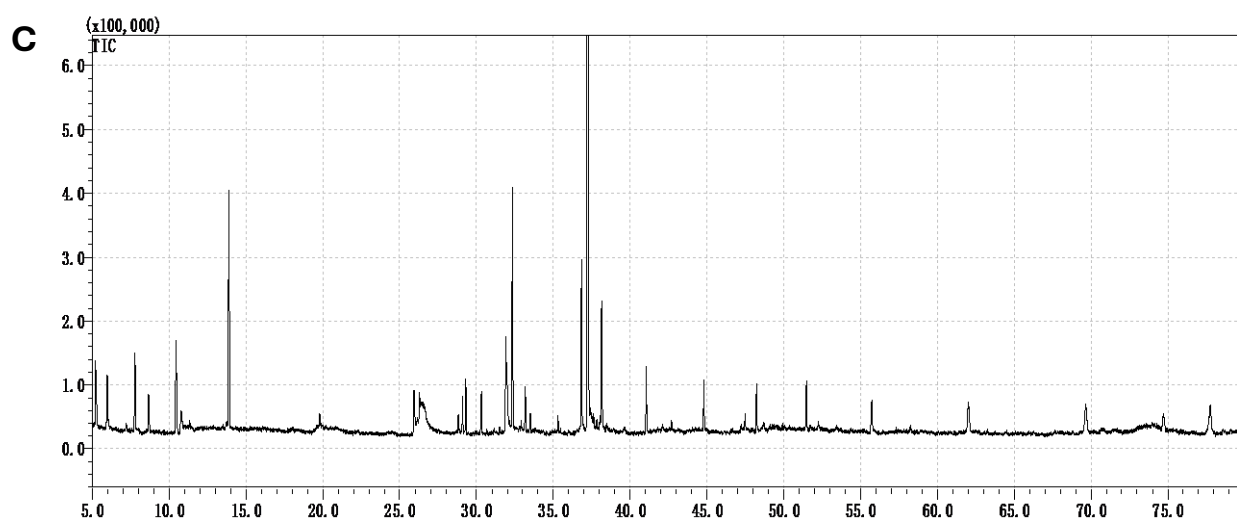

Supplement: Supplementary file 1 [file Supplementary_file_1.zip › Clean Revised Supplementary Figures_3.pdf]

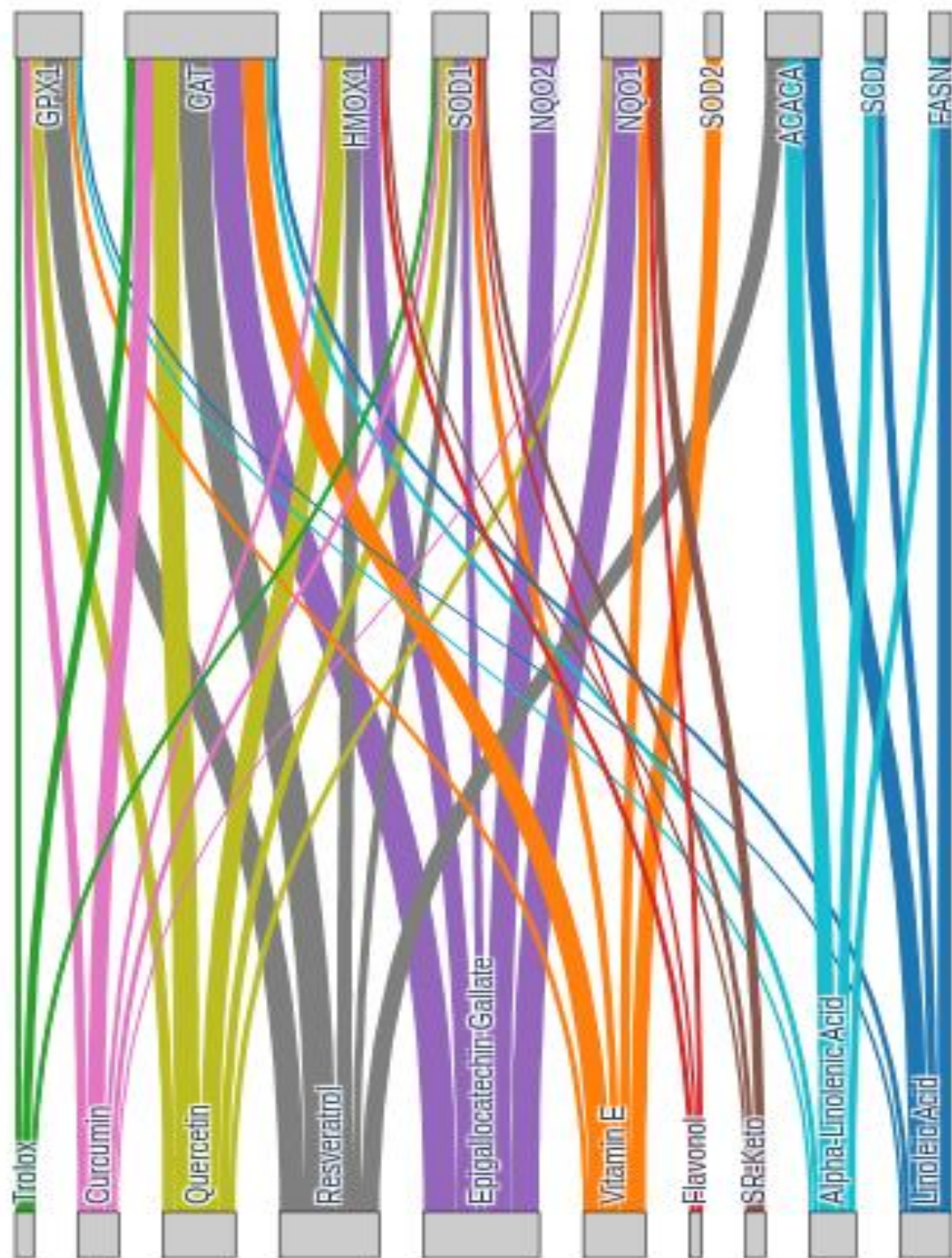

Supplement: Supplementary file 1 [file Supplementary_file_1.zip › Clean Revised Supplementary Figures_4.pdf]

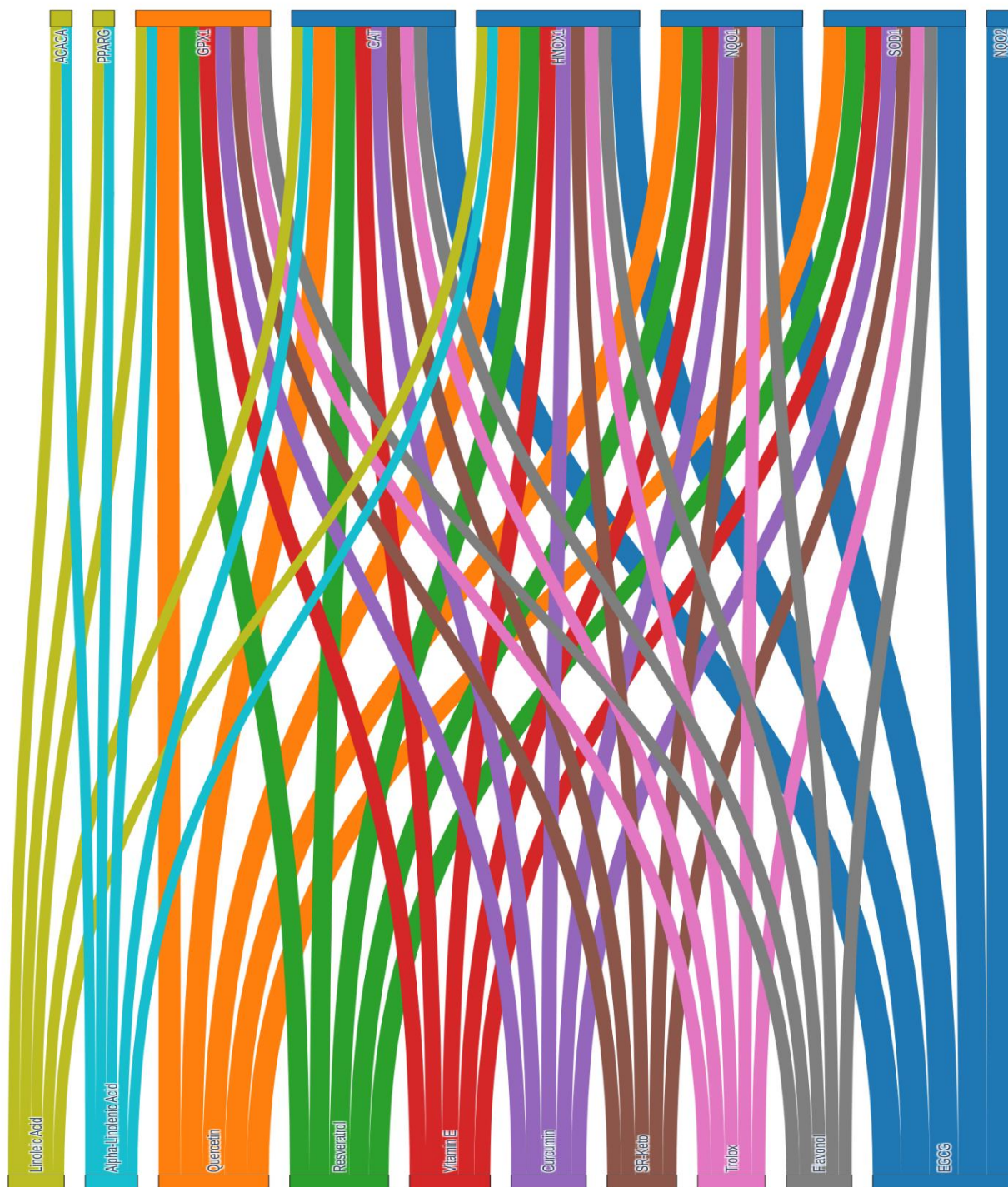

Supplement: Supplementary file 1 [file Supplementary_file_1.zip › Clean Revised Supplementary Figures_5.pdf]

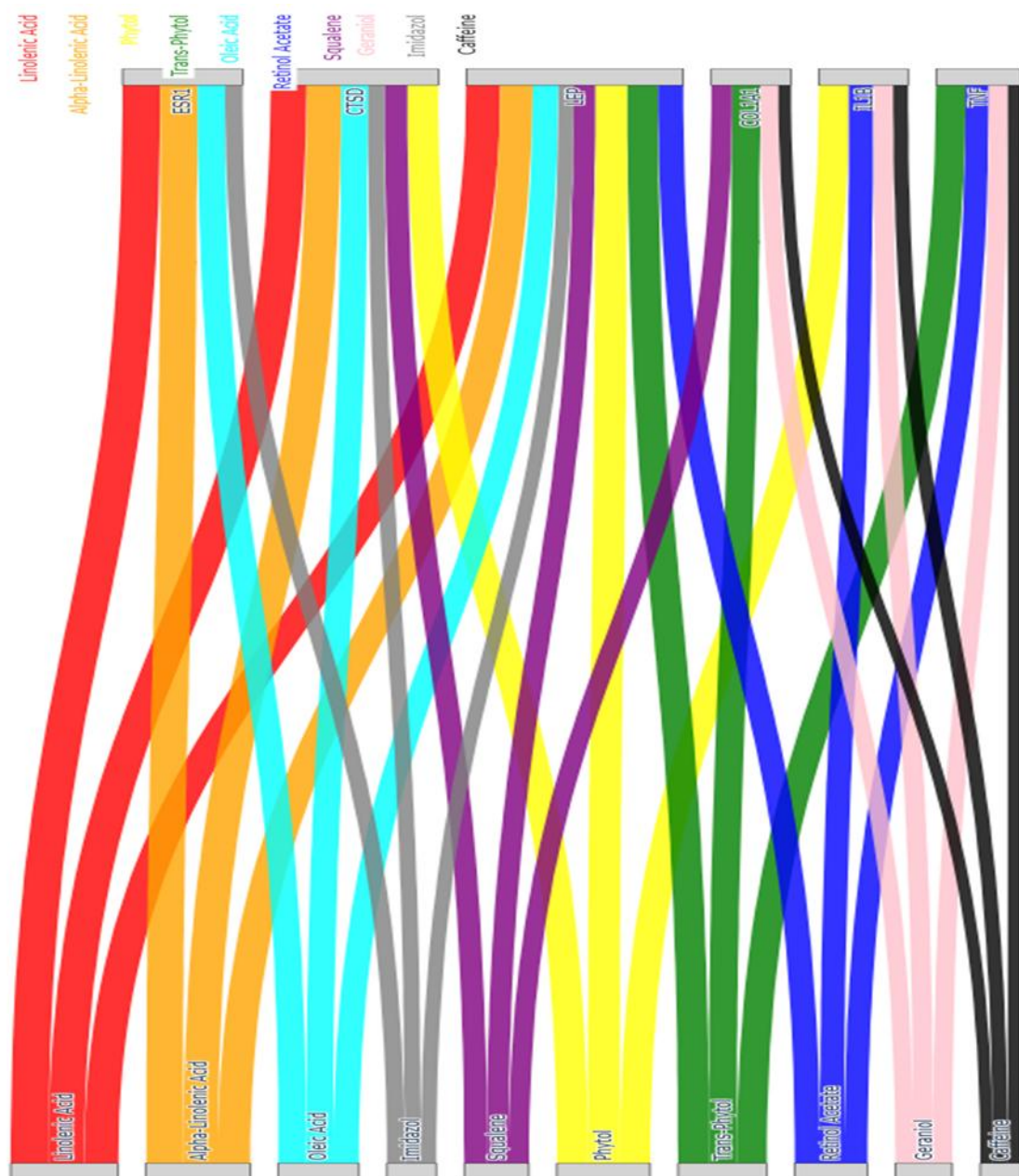

Supplement: Supplementary file 1 [file Supplementary_file_1.zip › Clean Revised Supplementary Figures_7.pdf]

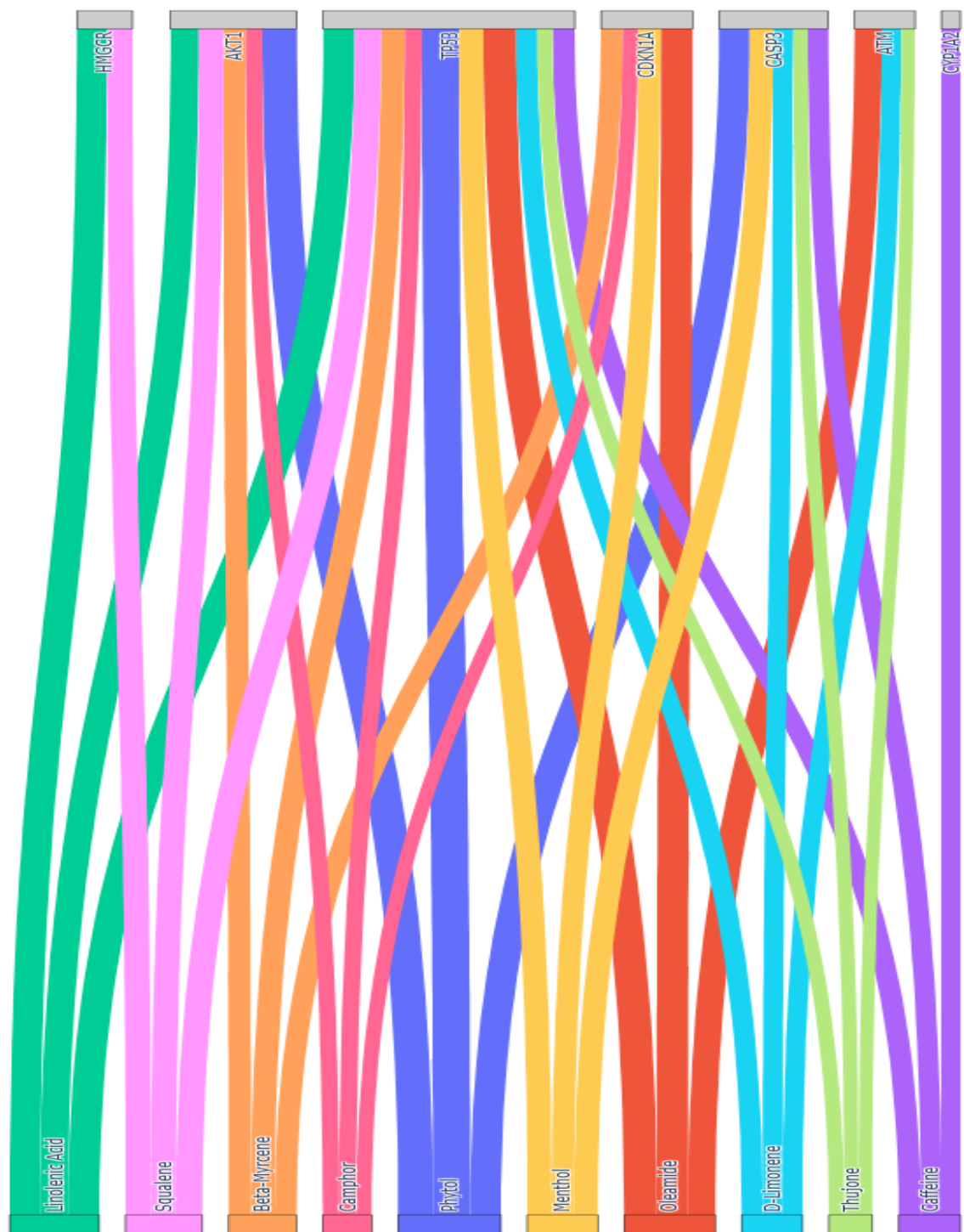

Supplement: Supplementary file 1 [file Supplementary_file_1.zip › Clean Revised Supplementary Figures_8.pdf]
