# Supplementary material for: Integrative phytochemical profiling and in silico nutrigenomic predictions of Chinese tea–Saudi Mentha longifolia blend formulations
Source: Front Nutr. 2026 Mar 27;13:1753616. doi: 10.3389/fnut.2026.1753616 (PMC13065726; doi:10.3389/fnut.2026.1753616)
Supplement: Supplementary file 1 [file Clean Revised Supplementary Figures_6.pdf › Clean Revised Supplementary Figures_6.pdf]

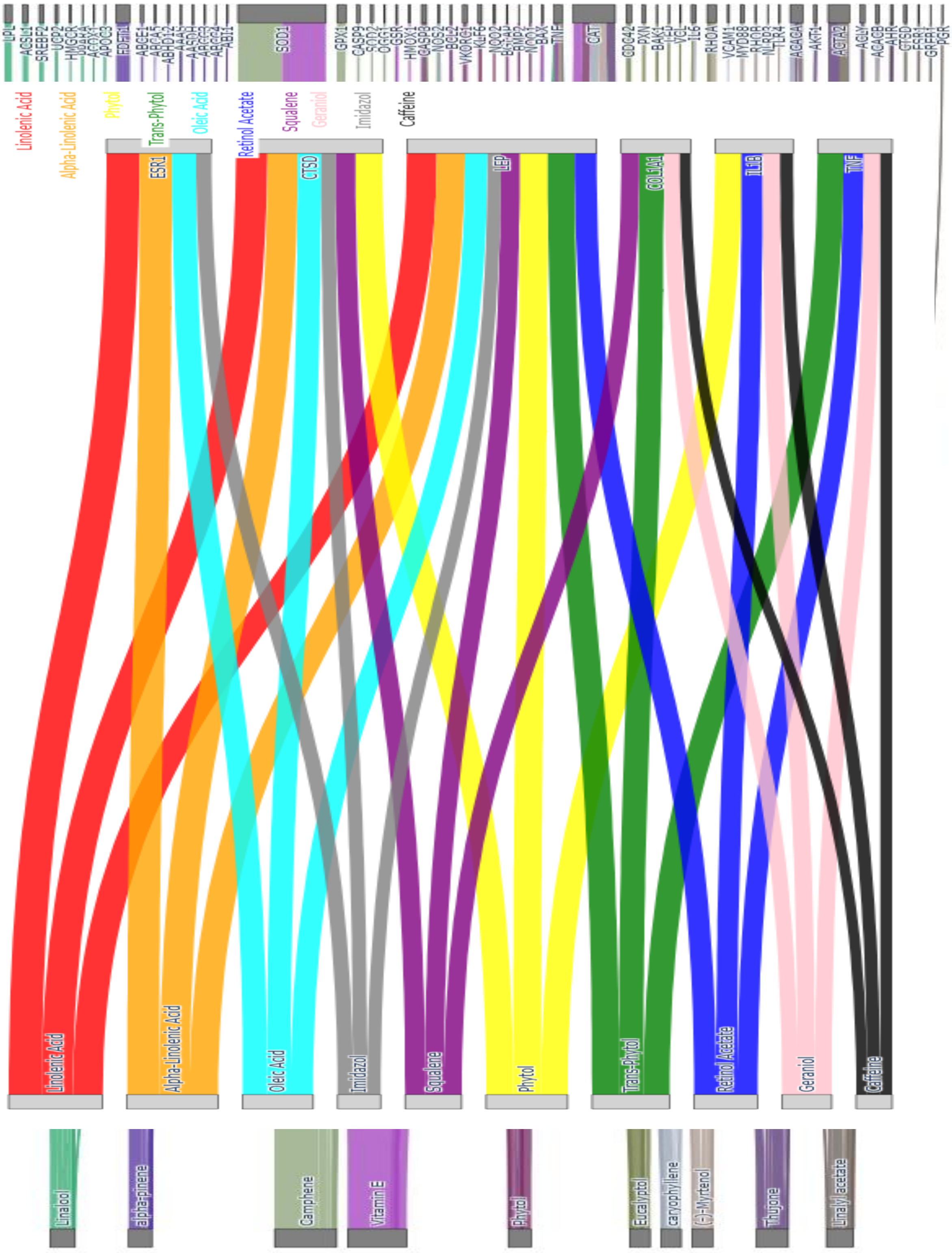
Linolenic Acid
Alpha-Linolenic Acid
Phytol
Trans-Phytol
Oleic Acid
Retinol Acetate
Squalene
Geraniol
Imidazol
Caffeine
ESR1
CTSD
LEP
COL1A1
IL1B
TNF
SOD1
CAT
Imidazol
Linalool
alpha-pinene
Camphene
Vitamin E
Eucalyptol
caryophyllene
Thujone
Linalyl acetate
